# Novel structured ADAM17 small-molecule inhibitor represses ADAM17/Notch pathway activation and the NSCLC cells’ resistance to anti-tumour drugs

**DOI:** 10.3389/fphar.2023.1189245

**Published:** 2023-06-29

**Authors:** Meng Chi, Yamin Jie, Ying Li, Duo Wang, Man Li, Dan Li, Mingyan E, Yongwu Li, Na Liu, Anxin Gu, Guanghua Rong

**Affiliations:** ^1^ Department of Anesthesiology, Harbin Medical University Cancer Hospital, Harbin, Heilongjiang, China; ^2^ Department of Radiation Oncology, The Fourth Affiliated Hospital of Harbin Medical University, Harbin, Heilongjiang, China; ^3^ Heilongjiang Provincial Key Laboratory of Hard Tissue Development and Regeneration, The Second Affiliated Hospital of Harbin Medical University, Harbin, China; ^4^ Heilongjiang Academy of Medical Sciences, Harbin, China; ^5^ Department of Neurology, The 2nd Affiliated Hospital of Harbin Medical University, Harbin, Heilongjiang, China; ^6^ Department of Endoscopy, Harbin Medical University Cancer Hospital, Harbin, Heilongjiang, China; ^7^ Department of Radiation Oncology, Harbin Medical University Cancer Hospital, Heilongjiang, China; ^8^ Department of Nuclear Medicine, The Fifth Medical Center of PLA General Hospital, Beijing, China; ^9^ Department of Oncology, The Fifth Medical Center of PLA General Hospital, Beijing, China

**Keywords:** non-small-cell lung cancer, antitumour drugs, small-molecule inhibitor, lung adenocarcinoma, lung squamous cell carcinoma

## Abstract

**Background and aims:** The outcomes of current treatment for non-small cell lung cancer (NSCLC) are unsatisfactory and development of new and more efficacious therapeutic strategies are required. The Notch pathway, which is necessary for cell survival to avert apoptosis, induces the resistance of cancer cells to antitumour drugs. Notch pathway activation is controlled by the cleavage of Notch proteins/receptors mediated by A disintegrin and metalloproteinase 17 (ADAM17); therefore, ADAM17 is a reliable intervention target for anti-tumour therapy to overcome the drug resistance of cancer cells. This work aims to develop and elucidate the activation of Compound **2b**, a novel-structured small-molecule inhibitor of ADAM17, which was designed and developed and its therapeutic efficacy in NSCLC was assessed via multi-assays.

**Methods and results:** A lead compound for a potential inhibitor of ADAM17 was explored via pharmacophore modelling, molecular docking, and biochemical screening. It was augmented by substituting two important chemical groups [R1 and R2 of the quinoxaline-2,3-diamine (its chemical skeleton)]; subsequently, serial homologs of the lead compound were used to obtain anoptimized compound (**2b**) with high inhibitory activity compared with leading compound against ADAM17 to inhibit the cleavage of Notch proteins and the accumulation of the Notch intracellular domain in the nuclei of NSCLC cells. The inhibitory activity of compound **2b** was demonstrated by quantitative polymerase chain reaction and Western blotting. The specificity of compound **2b** on ADAM17 was confirmed via point-mutation. Compound **2b** enhanced the activation of antitumor drugs on NSCLC cells, in cell lines and nude mice models, by targeting the ADAM17/Notch pathway.

**Conclusion:** Compound **2b** may be a promising strategy for NSCLC treatment.

## 1 Introduction

Lung cancer (LC) is a major cause of morbidity and mortality in humans (Sung et al., 2021; Byrnes et al., 2022; Li et al., 2022). LC comprises >50 histo-morphological subtypes; among these, non-small cell lung cancer (NSCLC) and small-cell lung carcinoma (SCLC) are most common (Herbst et al., 2018; Reck et al., 2022). NSCLC affects approximately 80%–85% of all patients with LC. Lung adenocarcinoma (LUAD) and lung squamous cell carcinoma (LSCC) account for approximately 40%–50% and 20%–30% of all NSCLC cases, respectively (Forde et al., 2022; Tan and Tan, 2022). Fewer than 40% of patients with NSCLC are diagnosed at an early stage (stages I or II) of the disease; hence, early diagnosis is necessary for considering tumour resection (Cooper et al., 2022; Li et al., 2022). More than 60% of patients with NSCLC initially diagnosed with locally advanced NSCLC or metastatic disease (stages III or IV) cannot undergo resection. Therefore, conventional chemotherapy and radiotherapy are the common treatment choices for these patients (Jonna and Subramaniam, 2019; Mithoowani and Febbraro, 2022). Recently, molecular-targeted drugs such as tyrosine kinase inhibitors (TKIs) and immune-checkpoint inhibitors (ICIs) are widely used. However, the overall efficacy of NSCLC treatment is unsatisfactory and the prognosis is poor (Jonna and Subramaniam, 2019; Mithoowani and Febbraro, 2022). Therefore, research and development of newer and more efficacious therapeutic strategies for NSCLC are required.

The Notch pathway regulates the proliferation, differentiation, and drug resistance of mammalian cells (Zhang et al., 2017; Osmani et al., 2018; Jia et al., 2021a). Notch pathway activation is directly coupled with the cleavage of the Notch protein/receptor to release the Notch intracellular domain (NICD) (Meurette and Mehlen, 2018; Yang et al., 2019a). Relocation of NICD from the cytoplasm to the nucleus mediates cell-fate decisions; NICD mediates expression of pro-survival, anti-apoptotic factors, and induces the epithelial–mesenchymal transition (EMT) of cells, eventually upregulating the resistance of cells to antitumour drugs (Meurette and Mehlen, 2018; Yang et al., 2019a). The Notch pathway is activated by a two-step cleavage of the Notch protein. The first, and significant, step is mediated by A disintegrin and metalloproteinase 17 (ADAM17). Approximately 30 ADAMs have been identified (Calligaris et al., 2021; Zhou et al., 2022). In human cancer (e.g., NSCLC) cells, inhibition of ADAM17 expression can inhibit the cleavage and activation of the Notch pathway, thereby enhancing the sensitivity of the cells to antitumour drugs (Zhang et al., 2018). In 2019, Yang et al. reported that micro-RNA-3163 enhances the sensitivity of hepatocellular carcinoma cells to anti-tumour drugs by targeting the 3′-untranslated region of ADAM17 (Jia et al., 2021a). However, an ADAM17-specific small-molecule inhibitor has not yet been developed. Therefore, exploring a specific inhibitor of ADAM17 is a rational approach in newer drug development.

In the present study, compound **2b** (a preferred compound)**,** a novel-structured small-molecule inhibitor of ADAM17, was designed and obtained for NSCLC treatment. Treatment with compound **2b** inhibited the cleavage and activation of Notch pathway and in addition, enhanced the sensitivity of NSCLC cells to antitumour drugs. Our results elucidate the role of ADAM17 in NSCLC and help provide more treatment options for NSCLC.

## 2 Materials and methods

### 2.1 Patients, clinical specimens, and database

A total of 79 pairs of NSCLC [including the LSCC, LUAD, large cell lung cancer (LCC)], and adjacent non-tumour (paired para-tumour) tissues were collected by the Department of Radiation Oncology, the Fourth Affiliated Hospital of Harbin Medical University from March 2018 to July 2020. These clinical specimens were obtained from the daily surgery with the written consent of the patients. The usage of all human related materials was approved by the Medical Ethics Committee of the Fourth Affiliated Hospital of Harbin Medical University (approval ID: KY 2021-02). The tissue specimens were stored in our lab as follows: 1) some were soaked in RNA later and frozen in liquid nitrogen; and 2) in the others, the total RNA was directly extracted and these RNA samples were frozen in liquid nitrogen. The pathologic subtypes of all tissue specimens were assessed and confirmed by pathological analysis (H&E staining). The diagnostic and inclusion and exclusion criteria of patients with NSCLC conformed to the Chinese Medical Association’s (2022 version) clinical diagnosis and treatment guidelines for lung cancer as follows: 1) patients with NSCLC were diagnosed by CT, MRI, or PET; 2) the head-neck and bone CTs and digestive system ultrasound-exploration were performed preoperatively; 3) only patients without severe obstruction of pulmonary function or stent placement within the past year (with a cardiac function >56%) were considered for surgery. The baseline information of patients with NSCLC are presented in Table 1. The sample size (79 paired NSCLC/non-tumour tissues) was considered to have adequate power for detecting a pre-specified effect size (the 1-β: 0.8; α/2: 0.025; *p* < 0.05) (González-Foruria et al., 2017) for the original/alternative hypotheses in the statistical analysis. According to the original hypothesis, the endogenous expression level of the targeting gene (including NOTCH1-4, or ADAM17) was hypothesised to have no significant difference between the non-tumour and tumour tissues. According to the alternative hypothesis, the expression level of these genes was hypothesised to have a significant difference between the non-tumour and tumour tissues.

**TABLE 1:** The baseline information of patients involved in the presence work.

Clinical characters	Values
Age	51 (32–69) (Median age [the upper and lower limit])
Gender	Male 65.82% (52/79)
Tumor stage	I stage 69.62% (55/79) (IA + IB)
	II stage 30.38% (24/79) (IIA + IIB)
Tumor number	1, 89.87% (71/79)
	2, 7.59% (6/79)
Tumor sites	Left lower lobe of Lung organs 12.66% (10/79)
	Left upper lobe of Lung organs 21.52% (17/79)
	Right lower lobe of Lung organs 15.29% (12/79)
	Right middle lobe of Lung organs 3.80 (3/79)
	Right upper lobe of Lung organs 46.84% (37/79)
Tumor location	Central 39.24% (31/79)
	Peripheral 60.76% (48/79)
Sub-types	Lung adenocarcinoma 62.03% (49/79)
	Large cell lung cancer 2.53% (2/79)
	Lung squamous carcinoma 35.44% (28/79)
Tumor sizes	1.2 (0.5–2.5 [diameter/cm]) (Median [upper and lower limit values])

The gene expression information by TCGA or GTEs databases were obtained from GEPIA 2 (Gene expression profiling interactive analysis) online tool graphical database (Yang et al., 2020). All gene expression information (including NOTCH1-4, ADAM17, and epidermal growth factor receptor [EGFR]) were matched with TCGA data and the results presented as TPM (transcript per million).

### 2.2 Cell lines, vectors, and anti-tumour drugs

The cell lines, A549 (LUAD), H460 (LCC), and H520 (LSCC), were purchased from the National Infrastructure of Cell Line Resources located at the Chinese Academy of Medical Sciences/China Union Medical College (Beijing, China). Cells were cryopreserved in liquid nitrogen and cultured in a Roswell Park Memorial Institute 1640 medium (RPMI-1640) containing 10% foetal bovine serum (FBS) at 37 °C in an atmosphere of 5% CO_2_.

TKIs (erlotinib, gefitinib, afatinib, osimertinib, and anlotinib) and chemotherapy agents (gemcitabine, epirubicin, docetaxel, and oxaliplatin) were purchased from Selleck Chemicals (Houston, TX, United States). The small molecular inhibitors for ADAM17, pratastat, terfenadine and ZLDI-8, were gifts form Prof. and Dr. Yingshi Zhang in Shenyang Pharmaceutical University. These TKIs and chemotherapy agents were prepared as formulations for cell-based assays or animal experiments. For cellular experiments, the pure solid powders of TKIs and two chemotherapy agents (gemcitabine and docetaxel) were dissolved in dimethyl sulfoxide (DMSO) (Yang et al., 2020). These solutions of the antitumour drugs were further diluted in Dulbecco’s Modified Eagle Medium (DMEM) without FBS. Epirubicin and oxaliplatin were directly solubilized in DMEM without FBS.

For the vectors used in the present study, a lentiviral vector constructed from human ADAM17 wild-type full-length or point mutation sequences and ADAM17 siRNA were purchased from GeneChem (Shanghai, China). A lentiviral vector constructed from NICD of Notch 1 was gifted by Prof Yingshi Zhang of the Shenyang Pharmaceutical University; this vector has been described in previous studies (Calligaris et al., 2021). Lipofectamine 3000 reagent was used for the transfection of the lentiviral vectors according to the manufacturer’s instructions (Invitrogen, Thermo Fisher Scientific Inc., United States). The lentivirus were generated by transfection of the 293T producer cell line with the lentiviral vector and Lentiviral Packaging Mix (System Biosciences, United States). The NSCLC cells were cultured and transfected with the lentivirus (approximately 10^9^ pfu of lentivirus was inoculated per 5 ×10^6^ cells) and screened with G418 for stable cell transfection.

### 2.3 Virtual screening of ADAM17-based small-molecule inhibitors and identification of lead compounds

The construction of a pharmacophore model and virtual screening of small-molecule inhibitors using molecular docking were based on the three-dimensional (3D) protein crystal structure of ADAM17 (Protein Databank (PDB) ID code: 2I47) (Jie et al., 2021). The 3D protein crystal structure of ADAM17 (PDB ID code: 2I47) was stripped of water molecules and ligands in Discovery Studio (BIOVIA, Dassault Systèmes, San Diego, United States), supplemented with non-intact amino-acid residues, and hydrotreated. ADAM17 mediates the hydrolysis of tumour necrosis factor-α cleavage; this aspect may aid in the development of ADAM17 inhibitors (Jie et al., 2021). Atapattu et al. have suggested that 4-({[4-(But-2-Yn-1-Yloxy)phenyl]sulfonyl}methyl)-1-[(3,5-Dimethylisoxazol-4-Yl)sulfonyl]-N-Hydroxypiperidine-4-Carboxamide is a ligand or small-molecule inhibitor that interacts with the amino-acid residues Lys 315, Thr 347, and Tyr 436 in the 3D protein crystal structure of ADAM17 (PDB ID code: 2I47) (Jie et al., 2021). The “Prepare Ligands” and “Dock Ligands” programs in Discovery Studio were used and the ‘semi-flexible virtual docking’ (CDOCKER) was selected as the molecular-docking mode. PyMOL (www.pymol.org) was used to visualise molecular docking. Subsequently, the 3D protein crystal structure of ADAM17 (PDB ID code: 2I47) was defined. H-acceptor features were retained after defining the binding pocket (“Define Site” program) and generating the feature elements (“Find Features” program) and clustering (“Cluster” program). Based on the aforementioned information, 190,000 molecules were downloaded from the ZINC library (http://zinc.docking.org). The molecules in this database have been used in *in vitro* experiments and their activity data reported. DruLiTo (https://en.freedownloadmanager.org/Windows-PC/DruLiTo-FREE.html) was employed for preliminary screening of the compounds. Screening was based on Lipinski’s rule of five principles: 0< log *p* < 5; molecular weight ≤500; 1≤ hydrogen-bond donors ≤5; 1≤ hydrogen-bond acceptors ≤10. Thus, approximately 76,000 compounds that met requirements were screened and into Discovery Studio. The “Build 3D Database” program was run to create a database of small molecules comprising these 76,000 compounds, named “Molecular data,” for subsequent pharmacophore screening. Using the “Search 3D Database” program, the database was input as “Molecular data,” Pharmacophore as “Model 3,” and the remaining settings were set as default for filtering. The value in the “FitValue” column in the screening report was used to evaluate the matching of small molecules and pharmacophores. A higher “FitValue” value indicated better matching between the small molecules and pharmacophores.

### 2.4 Optimization of the lead compound’s structure

A leading compound (N-(3-((furan-2-ylmethyl)amino)quinoxalin-2-yl)-4- methylbenzenesulfonamide) (Figure 2) of ADAM17-based small-molecule inhibitors was developed by virtual screening (Section 2.3). Based on the chemical structure of this lead compound [the parent ring of the benzopyrazine structure and possible substituted chemical groups (R1 and R2)], a series of derivatives (test compounds) were designed and chemically synthesized.

**FIGURE 1:**
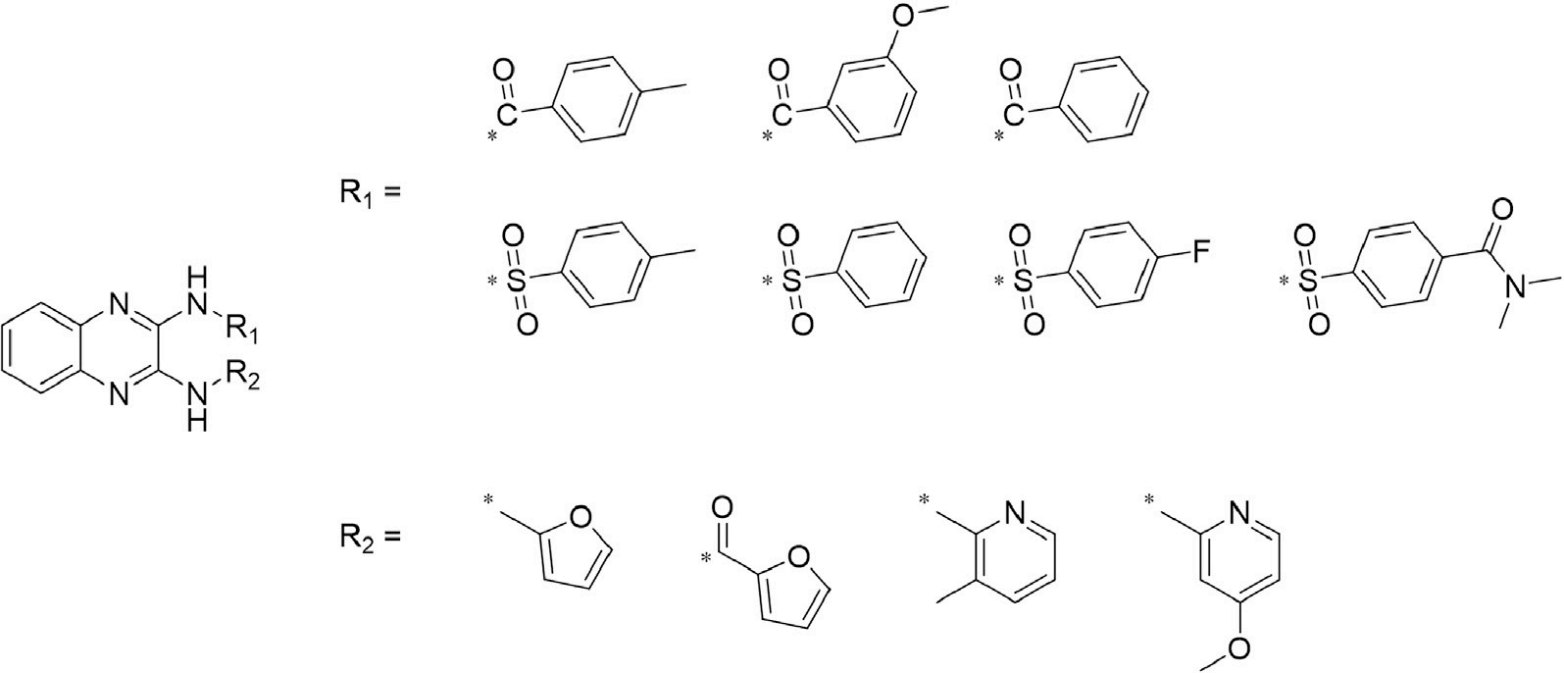
Core structure of the lead compound and the substitution of the R1 and R2 groups. Quinoxaline-2,3-diamine can be considered as the chemical skeleton of the leading compound (quinoxaline-2,3-diamine of Compound **2a**). R1 and R2 of this chemical skeleton can be a substitute for different chemical groups. R1 and R2 can have 7 substituents and 4 substituents, respectively.

**FIGURE 2:**
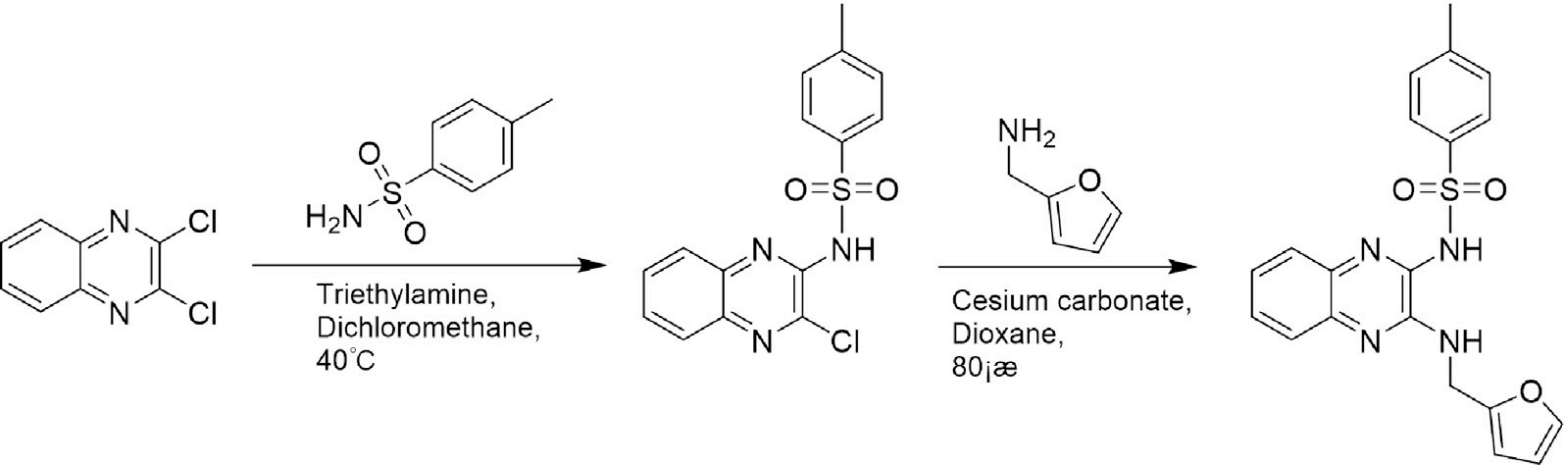
Synthetic route of a series of test compounds. The amino groups at the positions of R1 and R2 are first react to chlorine, and subsequently, react to the addition of the chemical substituent group of R1 or R2. Figure 2 showed Compound 2a as an example.

The R1 structure (Figure 1) retained the interaction between the carbonyl group and Leu 348 and maintained the π–π conjugation between the group and His 415/His 405. The R2 structure retained a hydrogen-bond acceptor to maintain the hydrogen-bond interaction with Thr 347 (Figure 1). Based on four R2 (i.e., compounds 1–4) and six R1 (Figure 1) structures, 20 compounds were synthesized. Thus, the synthetic route of the compound was determined (Figure 2). The leading compound in this series is compound **2a** and this synthetic route (Figure 2) is based on the example of lead compound: compound 2a.

A549 cells were treated with test compounds at a dose of 1 μmol/L when evaluating the activity of test compounds. Survivin expression downstream of Notch was detected by real-time reverse transcription-quantitative polymerase chain reaction (RT-qPCR). The activity of test compounds was determined based on the inhibition of survivin’s expression by the test compounds.

### 2.5 Molecular docking between compound 2b and ADAMs for binding energy analysis

Through online searching, only four ADAMs crystal structures have been clearly resolved for ADAM33, ADAM22, ADAM10, and ADAMTS-4, in addition to ADAM17. For this reason, to demonstrate the selectivity of compound 02b for ADAM17, the isoform structures of ADAM proteins that could be found in the PDB database (ADAM33, ADAM22, ADAM10, or ADAMTS-4) were screened here and the selectivity of 02b for ADAM17 was demonstrated by virtual docking between these four isoforms with compound 2b. The Autodock 4.2.6 software was used to virtually dock the compound 2b to the four ADAMs. During docking, only the conformation of the ligand (compound 2b) was changed, while the protein conformation was kept unchanged, and all other parameters were used as default values of the software. Then, the docking results were visualised and analysed by PyMOL 2.2.0 software. The magnitude of the binding energy provides evidence of the ability to bind between large and small molecules. A binding energy < −7.0 kcal/mol indicates a strong binding capacity.

This method was also used to measure the comparison between the different compounds acting on ADAM17 and to determine the strength of their action based on the binding capacity of these compounds with ADAM17.

### 2.6 RT-qPCR

Expression of related factors in NSCLC cells and tissues was measured by RT-qPCR, in accordance with Wang et al.’s description (Condon et al., 2007; Wang et al., 2021). RNA in NSCLC cells was directly extracted. For NSCLC tissues, RNA was extracted after grinding with liquid nitrogen. Samples of extracted RNA were reverse-transcribed and subsequently subjected to one-step qPCR. Expression of each factor was calculated according to the cycle threshold (Ct) values of each factor and β-Actin (housekeeping gene). Heat-maps were obtained according to the methods described by Zhou et al. and Ma et al. (Zhou et al., 2021; Ma et al., 2022). The reference-scale for heat map was inferred as folds of changes in the control group. Positive folds of changes indicated that the mRNA expression of target genes in cells of compound **2b** treatment group was increased relative to the control group; negative folds of changes indicated that the mRNA expression of target genes in cells of compound **2b** treatment group is decreased relative to the control group. The colour and the brightness of the heat map bands visually reflected the expression level of the target genes in each group. The primers used are listed in Table 2.

**TABLE 2:** The primers used in the qPCR assays.

Factors	Primers	Sequences
NICD	Forward Sequence	5′-CCG​ACG​CAC​AAG​GTG​TCT​T-3′
	Reverse Sequence	5′-GTC​GGC​GTG​TGA​GTT​GAT​GA-3
ADAM17	Forward Sequence	5‘-GGT​GGA​TGA​AGG​AGA​AGA​GTG​TGA-3’
	Reverse Sequence	5‘-CAG​CAG​GTG​TCG​TTG​TTC​AGA​TAC-3’
E-Cadherin (CDH1)	Forward Sequence	5′-CTC​CTG​AAA​AGA​GAG​TGG​AAG​TGT-3′
	Reverse Sequence	5′-CCG​GAT​TAA​TCT​CCA​GCC​AGT​T-3′
EGFR	Forward Sequence	5′-AAC​ACC​CTG​GTC​TGG​AAG​TAC​G-3′
	Reverse Sequence	5′-TCG​TTG​GAC​AGC​CTT​CAA​GAC​C-3′
NOTCH1	Forward Sequence	5′-GGT​GAA​CTG​CTC​TGA​GGA​GAT​C-3′
	Reverse Sequence	5′-GGA​TTG​CAG​TCG​TCC​ACG​TTG​A-3′
NOTCH2	Forward Sequence	5′-GTG​CCT​ATG​TCC​ATC​TGG​ATG​G-3′
	Reverse Sequence	5′-AGA​CAC​CTG​AGT​GCT​GGC​ACA​A-3′
NOTCH3	Forward Sequence	5′-TAC​TGG​TAG​CCA​CTG​TGA​GCA​G-3′
	Reverse Sequence	5′-CAG​TTA​TCA​CCA​TTG​TAG​CCA​GG-3′
NOTCH4	Forward Sequence	5′-TTC​CAC​TGT​CCT​CCT​GCC​AGA​A-3′
	Reverse Sequence	5′-TGG​CAC​AGG​CTG​CCT​TGG​AAT​C-3′
N-cadherin (CDH2)	Forward Sequence	5′-CCTGGAT CGCGAGCAGATA-3′
	Reverse Sequence	5′-CCA​TTC​CAA​ACC​TGG​TGT​AAG​AAC-3′
Vimentin	Forward Sequence	5′-ACC​GCA​CAC​AGC​AAG​GCG​AT-3′
	Reverse Sequence	5′-CGA​TTG​AGG​GCT​CCT​AGC​GGT​T-3′
Survivin (BIRC2)	Forward Sequence	5′-ACA​TGC​AGC​TCG​AAT​GAG​AAC​AT-3′
	Reverse Sequence	5′-GATTCCCA ACACCTCAAGCCA-3′
cIAP-1 (BIRC3)	Forward Sequence	5′-GTG​TTC​TAG​TTA​ATC​CTG​AGC​AGC TT-3′
	Reverse Sequence	5′-TGG​AAA​CCA​CTT​GGC​ATG​TTG​A-3′
cIAP-2 (BIRC5)	Forward Sequence	5′-CAA​GGA​CCA​CCG​CAT​CTC​T-3′
	Reverse Sequence	5′-AGC​TCC​TTG​AAG​CAG​AAG​AAA​CA-3′
β-actin (the loading control)	Forward Sequence	5′-CAC​CAT​TGG​CAA​TGA​GCG​GTT​C-3′
	Reverse Sequence	5′-AGGTCTTTGCGGA TGTCCACGT-3′
Twist	Forward Sequence	5′-GCC​AGG​TAC​ATC​GAC​TTC​CTC​T-3′
	Reverse Sequence	5′-TCC​ATC​CTC​CAG​ACC​GAG​AAG​G-3′
Snail	Forward Sequence	5′-TGC​CCT​CAA​GAT​GCA​CAT​CCG​A-3′
	Reverse Sequence	5′-GGGACA GGAGAAGGGCTTCTC-3′
GLUT1	Forward Sequence	5′-TTG​CAG​GCT​TCT​CCA​ACT​GGA​C-3′
	Reverse Sequence	5′-CAG​AAC​CAG​GAG​CAC​AGT​GAA​G-3′
HIF-1α	Forward Sequence	5′-TAT​GAG​CCA​GAA​GAA​CTT​TTA​GGC-3′
	Reverse Sequence	5′-CAC​CTC​TTT​TGG​CAA​GCA​TCC​TG-3′
EPAS-1 (HIF-2α)	Forward Sequence	5′-CTGTGT CTG​AGA​AGA​GTA​ACT​TCC-3′
	Reverse Sequence	5′-TTG​CCA​TAG​GCT​GAG​GAC​TCC​T-3′

### 2.7 Cell experiments, cellular sub-faction, and Western blotting

Experiments were performed according to Du et al.’s method (Ma et al., 2020). Post-culture, the NSCLC cells were treated with solvent (control) or drugs and lysed to produce nuclear and cytoplasmic fractions. These fractions were separated by two-step centrifugation at 25°C temperature (nuclear and cytoplasmic fractions at 800 revolutions per minute (rpm) for 3 min and 12,000 rpm for 5 min, respectively) and analysed via Western blotting. Protein expression of NICD or ADAM17 in these fractions was measured using their antibodies via Western blotting. Lamin-A and β-Actin were used as the nuclear and cytoplasmic indicators, respectively. The Western blot images were quantitatively analysed by ImageJ software [National Institutes of health (NIH), Bethesda, Maryland, United States]. The Western blot image was adjusted to pure black and white and subsequently, the protein strip was circled and quantified by the total area multiplied by the grey level. Regarding the antibodies used in the present study, the NICD’s antibody was gifted by Prof Yingshi Zhang of the Shenyang Pharmaceutical University (it has been used in some previous studies) (Calligaris et al., 2021), while the antibodies for β-Actin, GAPDH, or Lamin A were purchased from Abcam Corporation, United Kingdom.

### 2.8 Action of anti-tumour drugs on NSCLC cells

NSCLC cells were cultured and prepared as a single-cell suspension. They were seeded into a 96-well cell culture plate at approximately 8,000 cells/well. After cells had become fully adherent, the supernatant was discarded and replaced with a working solution of the drugs. The dose of compound **2b** was 10, 1, or 0.1 μmol/L. The dose of each antitumour drug is listed in Table 3. Cells were treated with the antitumour drugs (compound **2b** alone for 48 h, or pretreatment with compound **2b** for 4–6 h, followed by antitumour drug for 40–48 h). Subsequently, the cells were treated with 3-(4,5-Dimethylthiazol-2-yl)-2,5-diphenyltetrazolium bromide (MTT; 50 mmol/L) for 5–6 h. Cell samples were lysed with DMSO and analysed by a multifunctional full-wavelength microplate reader to measure the absorbance at 490 nm (Du et al., 2021; LiFengJiaJiangCaoWeiZhangLu, 2021) and the half-maximal inhibitory concentration (IC_50_) of antitumour drugs was calculated (Du et al., 2021; LiFengJiaJiangCaoWeiZhangLu, 2021).

**TABLE 3:** The concentrations of Anti-tumor drugs in the cell-based assays.

Anti-tumor drugs	Concentrations (μmol/L)						
Erlotinib	10	3	1	0.3	0.1	0.03	0.01
Gefitinib	10	3	1	0.3	0.1	0.03	0.01
Afatinib	10	3	1	0.3	0.1	0.03	0.01
Osimertinib	10	3	1	0.3	0.1	0.03	0.01
Anlotinib	3	1	0.3	0.1	0.03	0.01	0.003
Oxaliplatin	3	1	0.3	0.1	0.03	0.01	0.003
Docetaxel	0.3	0.1	0.03	0.01	0.003	0.001	0.0003
Epirubicin	1	0.3	0.1	0.03	0.01	0.003	0.001
Gemcitabine	3	1	0.3	0.1	0.03	0.01	0.003

For drug synergy assays, synergy effect of drug pairs was quantitative defined by the Chou-Talalay equation. Calculation of the synergy index for the action of compound 2b and Gefitinib: 7 doses of compound 2b (0.03 μmol/L, 0.1 μmol/L, 0.3 μmol/L, 1 μmol/L, 3 μmol/L, 10 μmol/L and 30 μmol/L) with 7 doses of Gefitinib (0.003 μmol/L, 0.01 μmol/L, 0.03 μmol/L, 0.1 μmol/L, 0.3 μmol/L, 1 μmol/L, and 3 μmol/L) were combined for A549 treatment. The IC50 values of Gefitinib in A549 cells with each of the seven doses of compound 2b was calculated. The synergistic index of the two effects was also calculated. Combinational Index (CI) < 1 indicates synergism; CI > 1 indicates antagonism (Tang et al., 2017). MDA-MB-231 and HCC1937 cells were treated with different concentrations of two single drugs and combinational treatment for 72 h, respectively, and cell viability was validated with CCK-8 kit according to the manufacturer’s instructions (Dojindo Laboratories, Japan).

### 2.9 *In vivo* tumour models

The animal experiments were performed in accordance with the Heilongjiang Provincial Key Laboratory of Hard Tissue Development and Regeneration protocol. The protocol for animal experiments (including the purchase, preservation, transportation, feeding, animal welfare, and the experimental design) was approved by the Animal Care and Use Committee of the Second Affiliated Hospital of Harbin Medical University (Harbin City, Heilongjiang Province, China) (approval number: SYDW-2022-082). In animal experiments, all results were obtained in accordance with the United Kingdom Animals (Scientific Procedures) Act, 1986 and associated guidelines. Nude mice (5 weeks old) were purchased from Si-Bei-Fu Corporation (Beijing, China). The base diet formula for experimental animal feeding was the AIN-93M (American Institute of Nutrition-1993 Maintenance), which provided the required nutrient levels for the long-term feeding of rodents. The nude mice were kept in SPF (specific pathogen free) conditions, all feed and bedding were pre-sterilized by ^60^Co-γ irradiation, and sterilized pure water was provided for drinking.

#### 2.9.1 Subcutaneous tumour model in nude mice

Nude mice were subcutaneously injected with cultured A549 cells prepared as a single-cell suspension. The pure powder of the drug was prepared as a formulation suitable for oral administration according to the methods described by Jiang et al. and Mao et al. (Feng et al., 2020; Jiang et al., 2021) A549 cells obtained by culture from the bottom of the culture dish were digested using trypsin, and re-suspended with sterilized PBS; during this procedure, the cells were washed thrice using PBS (cells were collected by centrifugation at 800 rpm for approximately 3 min at room temperature and subsequently re-suspended using PBS for one wash). Eventually, a 2.5 × 10^7^/mL cell suspension was prepared (using only PBS, without the addition of any Matrigel). The cell suspension was subcutaneously injected in nude mice using a sterilized disposable syringe, one site per animal, with a volume of 200 μL per injection, and approximately 5 × 10^6^ A549 cells per animal. The injection site injection was in the medial inguinal region adjacent to the femoral vein; this ensured that the needle did not disrupt the venous structure and allowed the injected fluid to surround the femoral vein. After 3–4 days, the injected suspension was gradually absorbed and further administration was performed.

The nude mice were orally administered antitumour drugs. In specific experiments, anlotinib or compound **2b** alone or in combination was administered every 2 days. During this procedure, the nude mice were orally administered a formulation of the drug. They were divided into the untreated (control), compound **2b** alone, anlotinib alone, and anlotinib combined with compound **2b** groups. In terms of dosage, animals in the control and compound **2b** treatment groups were orally administered solvent control and 1 mg/kg compound **2b,** respectively. Three doses of anlotinib, high (0.5 mg/kg), medium (0.2 mg/kg), and low (0.1 mg/kg), were administered with solvent control for the anlotinib alone group. High (0.5 mg/kg), medium (0.2 mg/kg), and low (0.1 mg/kg) doses of anlotinib were administered together with compound **2b** (1 mg/kg) for the anlotinib combined with compound **2b** group. After 20–25 days, the mice were euthanised and tumour tissue to calculate and measure the tumour volume and weight, respectively, was collected. RNA samples were extracted from tumour tissues. RT-qPCR was performed to measure the expression of Notch pathway-related factors.

#### 2.9.2 *In vivo* imaging of nude mice

We created an intra-lung tumour model using nude mice. The A549 cells were stably transfected with luciferase-enhanced green fluorescent protein vectors (by lentivirus), and injected through the mice’s tail vein. For the metastasis model, 2 × 10^5^ A549 cells were injected. Post-injection, the nude mice were orally administered the antitumour. In specific experiments, anlotinib or compound **2b** alone or in combination were administered every 2 days. The nude mice were administered an oral formulation of the drugs. They were divided into the untreated (control), compound **2b** alone, anlotinib alone, and anlotinib combined with compound **2b** groups. In terms of dosage, animals in the control and compound **2b** groups were orally administered solvent control and 1 mg/kg compound **2b,** respectively. The dosage of anlotinib alone was 0.2 mg/kg. Anlotinib 0.2 mg/kg dose was administered with compound **2b** 1 mg/kg dose for the anlotinib combined with compound **2b** group.

The metastasis of A549 cells in nude mice were measured by 1) live imaging and 2) pathological analysis of the lung tissue. In combination with the aforementioned experimental steps (tail-vein injection of cells and drug administration), small animal *in vivo* imaging was performed once after the A549 cells were injected (0-time point) and once after the treatment was completed (end-time point). In the *in vivo* imaging experiments, the nude mice were first anesthetized using an inhalation anaesthesia machine (initial anaesthesia with 1.5% [v/v] isoflurane and continuous anaesthesia with 0.5% [v/v] isoflurane), after which each mouse was intra-peritoneally injected with D-fluorescein (150 mg/kg). Five minutes later, the *in vivo* imaging was performed by using a Xenogen IVIS^®^ 2000 Luminal Imager (Xenogen, United States). Image data were acquired and measured using area analysis and Living Image^®^ Software (Xenogen, United States). Images were normalized according to colour intensity on each data set to quantify the lesions in the lung region. The A549 cells-induced lesions or nodules in the lung tissues of the nude mice were measured using the method described by Jiang et al. and Mao et al. *via* pathological staining (Feng et al., 2020; Jiang et al., 2021). The lung tissues were paraffin-embedded and sectioned separately; thus, the final paraffin sections were obtained. Subsequently, these paraffin sections were mounted on Plus slides and dried in an oven at 60°C and were placed on a Leica BondMax Immunostainer. Thereafter, eosin staining was performed. After staining, the slides were dehydrated and cover-slipped with Cytoseal 60 (Richard-Allan Scientific) mounting medium. Subsequently, the results of pathological staining were analysed by ImageJ software based on Shao et al.’s methods. The extent of all tissues and circled lesions were separately determined, and the proportion of lung tissues occupied by lesions was determined according to the total number of pixels in the total circled area; finally, quantitative analysis was performed.

#### 2.9.3 Cellular sub-fraction experiments

For selected subcutaneous tumour tissues, surgery was performed in an ultra-clean table to remove the tumour tissue from the subcutaneous area of the animal, after which the tumour tissue was ground using DMEM supplemented with 20% FBS, pre-sterilised 200 mesh steel sieve, and finally a single cell suspension was obtained. The cells were then subjected to nucleoplasmic separation to determine the distribution of ADAM17 and NICD in the nucleoplasm of the cells.

### 2.10 Statistical analyses

Statistical analyses were performed using Prism 8.0 (GraphPad, San Diego, CA, United States). The *IC*_*50*_ values of the antitumour drugs were calculated using Origin 6.0 (OriginLab, Northampton, MA, United States). All statistical analyses were performed using the GraphPad 8.0 (GraphPad Software Corporation, Armonk, NY, United States). For two groups/between-group comparisons, the data were first evaluated in terms of normal distribution (if the F value > 0.05, the data conformed to the normal distribution, and the unpaired *t*-test was used for testing; if the F value < 0.05, the data did not conform to the normal distribution and the rank sum test was used). Since the two sets of data were independent samples, and not paired samples, and both sets of samples conformed to a normal distribution, the unpaired *t*-test was used to compare the difference in means. For multi-groups analysis, the one-way analysis of variance (ANOVA) and multiple comparison methods were used. The ANOVA was used to examine whether any difference between groups could be obtained. A *p*-value < 0.05 was considered statistically significant.

## 3 Results

### 3.1 Endogenous level of ADAM17 or Notch isoforms in NSCLC clinical specimens

**FIGURE 3:**
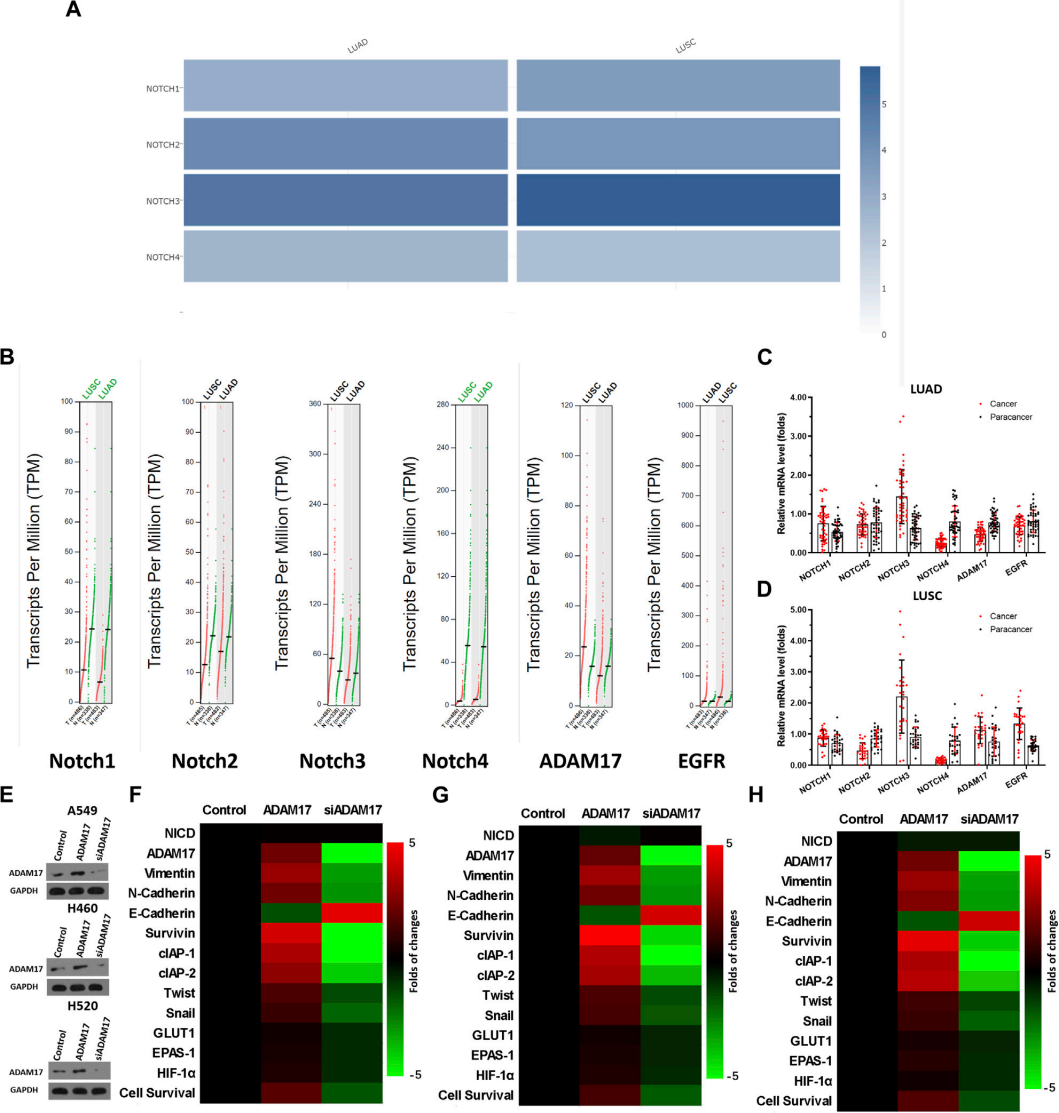
Clinical expression data of Notch and ADAM17 and confirmatory study of ADAM17 targets in NSCLC **(A–D)** Relevant data in TCGA and GTEx databases are retrieved from the graphical database GEPIA2, analysed, and plotted. **(A)** Expression levels of NOTCH1, NOTCH2, NOTCH3, and NOTCH4 in LUAD or LUSC in TCGA and GTEx databases, respectively, with results are exhibited as heat maps (cross-sectional comparison of NOTCH1-4). **(B)** Expression levels of NOTCH1, NOTCH2, NOTCH3, NOTCH4, ADAM17, and EGFR in LUAD and its corresponding non-tumour tissues or LUSC and its corresponding non-tumour tissues in TCGA and GTEx databases, results are exhibited as scatter plots with median (comparison of each gene in tumour-non-tumour tissues). **(C, D)** The expression of NOTCH1, NOTCH2, NOTCH3, NOTCH4, ADAM17, and EGFR is detected separately in 79 pairs of NSCLC/non-tumour tissues collected by our group *via* RT-qPCR. These 79 specimens included 49 specimens of adenocarcinoma **(C)** of the lung and 28 specimens of squamous lung cancer **(D)** respectively. The results are displayed as a scatter plot-bar chart **(B–D)**. **(E)** Overexpression or knockdown of ADAM17 in A549, H460, and H520, and the expression levels of ADAM17 in these cells are detected by Western blot **(F–H)** The expression level of ADAM17, and Notch pathway’s downstream genes are examined by RT-qPCR in A549 **(F)**, H460 **(G)** or H520 **(H)** and these results are exhibited as heatmaps according to relative mRNA expression. *p < 0.05.

Previous studies reported that the Notch pathway is crucial in NSCLC and is a promising therapeutic target for NSCLC treatment (McCoach et al., 2018; Sharif et al., 2020; Li et al., 2021; Mao et al., 2022); however, some publications and data from databases revealed that Notch may have a relatively lower expression in NSCLC tissues compared with normal tissues. The expression data of Notch isoforms in databases and clinical specimens were examined for scientific rigor and better clarification. As illustrated in Figure 3, the expression levels of four Notch isoforms, namely, NOTCH1∼4 in NSCLC and para-tumour non-tumour tissues are not consistent (Figure 3A). The median expression of NOTCH1 in LUAD and LUSC tumour tissue was lower than that in para-tumour tissues (Figure 3B). The expression trend of NOTCH2 was similar to that of NOTCH1 (Figure 3B). However, the expression of NOTCH3 in LUAD was significantly higher than that in para-tumour non-tumour tissues, and its median expression in LUSC was close to that in para-tumour non-tumour tissues (Figure 3B). The expression level of NOTCH4 in LUAD or LUSC was significantly lower than that in para-tumour tissues (Figure 3B). Moreover, the expression level of EGFR, an extremely well-recognized target for therapeutic intervention in NSCLC, in databases was examined, as a reference. As illustrated in Figure 3B, in NSCLC, the median value of EGFR’s expression in either LUAD or LUSC is approximately 10–20 TPM (transcript per million) (Figure 3B). This is comparable to the expression level of Notch, which is of a similar magnitude (Figure 3B). Notably, the results included in the TGCA or GTEs database revealed that most EGFR tissues have expression levels <100 TPM (Figure 3B); moreover, Notch isoforms are expressed at high levels in some NSCLC tissues (in particular, NOTCH1, NOTCH2, and NOTCH3’s expression could reach 60–90, 50–500 and 240–300 TPM, respectively) (Figure 3B). Similar results were observed when the results of the clinical specimens were compared with these data from the database (Figures 3C, D). Therefore, the expression level of Notch protein varies among different isoforms and Notch is not always less expressed in NSCLC tissues than in para-tumour tissues. Compared with EGFR’s data, Notch is clearly expressed in NSCLC; although this expression is generally high, it does not affect its use as a therapeutic target in NSCLC.

Although previous studies indicated that ADAM17 was a promising therapeutic target for NSCLC treatment (Baumgart et al., 2010; Kang et al., 2013; Ni et al., 2013; Yang et al., 2019b; Saad et al., 2019), the expression data of ADAM17 in the database and our clinical specimens were examined for scientific rigor and better clarification. As illustrated in Figure 3B, the median expression of ADAM17 is much higher in LUSC tissues than in the non-tumour tissues; furthermore, the expression levels were as high as 60–100 TPM in some LUSC tissues. The median expression of ADAM17 in LUAD was slightly lower than that in non-tumour tissues; furthermore, these levels were as high as 40–80 TPM in some LUAD tissues (Figure 3B). The expression of ADAM17 was also examined in the clinical specimens (Figures 3C, D). Accordingly, the function of ADAM17 was further confirmed by its overexpression or knockdown in NSCLC cells. As illustrated in Figure 3E, overexpression of ADAM17 in A549, H460 or H520 cells upregulated each drug resistance gene downstream of the Notch pathway, while knockdown of ADAM17 downregulated each drug resistance gene downstream of the Notch pathway (Figures 3F–H). Moreover, the overexpression or knockdown of ADAM17 did not affect NICD expression level (Figures 3F–H). The results, presented in the Tables, reveal that overexpression of ADAM17 in A549, H460 or H520 cells upregulated the resistance of NSCLC cells to antitumour drugs when the *IC*_*50*_ value of antitumour drugs on the cells was significantly increased (Table 4); knockdown of ADAM17 downregulated the resistance of NSCLC cells to antitumour drugs when the *IC*_*50*_ value of antitumour drugs on the cells was significantly decreased (Table 4). These results confirmed the role of ADAM17 in NSCLC (i.e., target validation was performed).

**TABLE 4:** Overexpression of ADAM17 or ADAM17 modulates the sensitivity of NSCLC cells to antitumor drugs.

Cell lines	Antitumor drugs	Control	ADAM17	siADAM17
		*IC*_*50*_ values (μmol/L)		
A549	Erlotinib	0.75 ± 0.42	5.50 ± 0.83	0.14 ± 0.03
	Gefitinib	1.03 ± 0.53	6.98 ± 0.26	0.36 ± 0.06
	Afatinib	0.94 ± 0.30	4.71 ± 0.20	0.15 ± 0.10
	Osimertinib	0.65 ± 0.11	3.16 ± 0.54	0.23 ± 0.05
	Anlotinib	0.49 ± 0.23	5.72 ± 0.59	0.10 ± 0.01
	Oxaliplatin	0.31 ± 0.14	5.50 ± 0.72	0.10 ± 0.02
	Docetaxel	0.06 ± 0.00	0.33 ± 0.08	0.01 ± 0.00
	Epirubicin	0.30 ± 0.15	1.62 ± 0.28	0.09 ± 0.02
	Gemcitabine	0.26 ± 0.10	0.51 ± 0.33	0.13 ± 0.04
H460	Erlotinib	0.96 ± 0.64	3.99 ± 0.25	0.36 ± 0.08
	Gefitinib	0.85 ± 0.50	7.89 ± 0.66	0.27 ± 0.18
	Afatinib	1.14 ± 0.91	4.35 ± 0.78	0.21 ± 0.02
	Osimertinib	0.93 ± 0.45	5.12 ± 0.77	0.28 ± 0.09
	Anlotinib	0.74 ± 0.21	2.19 ± 0.40	0.10 ± 0.00
	Oxaliplatin	0.53 ± 0.16	4.41 ± 0.62	0.23 ± 0.04
	Docetaxel	0.14 ± 0.07	0.55 ± 0.10	0.03 ± 0.01
	Epirubicin	0.75 ± 0.31	3.83 ± 0.49	0.36 ± 0.09
	Gemcitabine	0.37 ± 0.08	2.48 ± 0.94	0.06 ± 0.02
H520	Erlotinib	0.82 ± 0.25	3.88 ± 1.25	0.25 ± 0.03
	Gefitinib	0.79 ± 0.21	4.67 ± 0.98	0.12 ± 0.04
	Afatinib	0.91 ± 0.35	5.10 ± 0.61	0.32 ± 0.07
	Osimertinib	0.55 ± 0.23	4.73 ± 0.88	0.19 ± 0.08
	Anlotinib	0.43 ± 0.51	5.84 ± 0.96	0.16 ± 0.10
	Oxaliplatin	0.75 ± 0.34	5.31 ± 0.27	0.20 ± 0.25
	Docetaxel	0.08 ± 0.05	0.33 ± 0.13	0.01 ± 0.00
	Epirubicin	0.63 ± 0.25	3.64 ± 0.85	0.22 ± 0.05
	Gemcitabine	0.32 ± 0.06	1.84 ± 0.47	0.17 ± 0.03

### 3.2 Structural optimization of lead compounds

**FIGURE 4:**
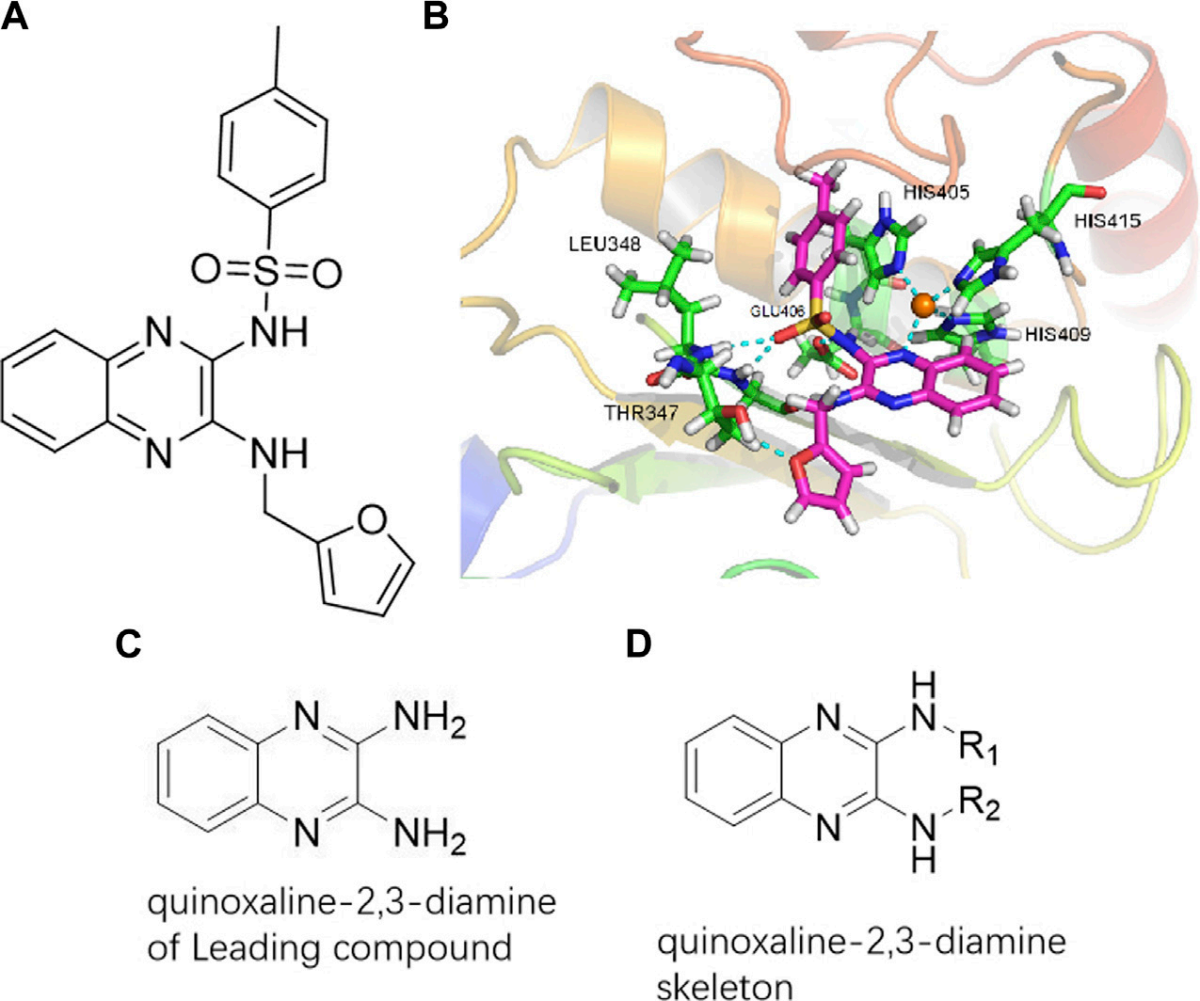
Lead compound of ADAM17’s small molecular inhibitor with a novel structure **(A)** The chemical structure of the leading compound: Compound **2a** (N-(3-((furan-2-ylmethyl)amino) quinoxalin-2-yl)-4-methylbenzenesulfonamide). The potential interaction of ADAM17-based small-molecule inhibitors (lead compound) with ADAM17 *via* molecular-docking. The key amino-acid disability ‘His 405, His 415, His 409, Thr 347, or Leu 348’ in ADAM17 that interacts with the inhibitors are exhibited. The chemical structure of the lead compound **(B)** and R1/R2 groups **(C)**.

To explore an effective inhibitor of ADAM17, a lead compound: compound 2a (N-(3-((furan-2-ylmethyl)amino) quinoxalin-2-yl)-4-methylbenzenesulfonamide) was identified *via* multi-assay screening (Figure 4A). The molecular docking between ADAM17 and the lead compound that explores the potential mechanism of lead compound on ADAM17, tis exhibited in Figure 4B. The parent ring (benzopyrazine) of the lead compound was placed in the hydrophobic cavity of ADAM17 and nitrogen atoms. His 405, His 409, and His 415 formed a stable triangular structure around Zn^2+^ through a monodentate coordination bond. Thr 347 and Leu 348 formed hydrogen bonds with compound branches, and this intermolecular interaction maintained the stable combination of the lead compound (Figure 4B).

A series of R1 and R2 structural fragments were designed based on the parent ring (benzopyrazine) of the lead compound (Figures 4C, D). Seven R1 and four R2 fragment structures were designed. The R1 and R2 fragments were combined and 20 testing compounds were synthesized (Figures 4C, D). Among these, compound **2b** most potently inhibited survivin expression (Table 5). Of particular note, the activity of compound **2b** was superior to that of leading compound 2a (Table 5). Thus, compound **2b** was considered the optimal compound for subsequent experiments.

**TABLE 5:** The inhibitory rates of compounds on Survivin’s expression .

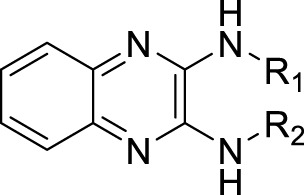			
No.	R1	R2	IR
01	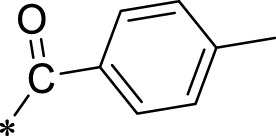	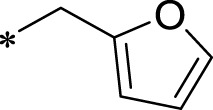	33.50 ± 2.05
4-methyl-N-(3-((3-methylpyridin-2-yl)amino)quinoxalin-2-yl)benzamide			
01a	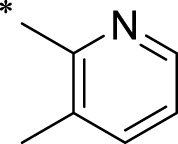	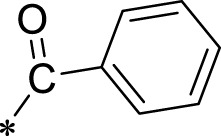	41.78 ± 2.60
4-methyl-N-(3-((3-methylpyridin-2-yl)amino)quinoxalin-2-yl)benzenesulfonamide			
01b	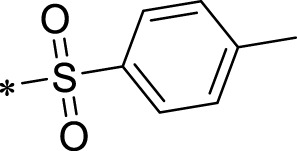	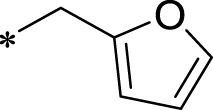	53.61 ± 2.64
N-(3-((3-methylpyridin-2-yl)amino)quinoxalin-2-yl)benzenesulfonamide			
01c	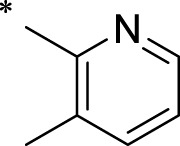	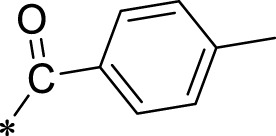	32.92 ± 3.22
4-fluoro-N-(3-((3-methylpyridin-2-yl)amino)quinoxalin-2-yl)benzenesulfonamide			
01d	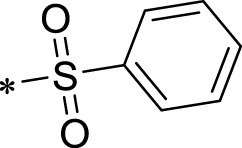	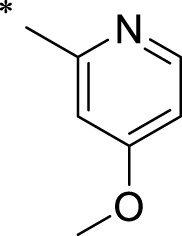	37.55 ± 0.38
N,N-dimethyl-4-(N-(3-((3-methylpyridin-2-yl)amino)quinoxalin-2-yl)sulfamoyl)benzamide			
01e	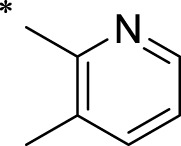	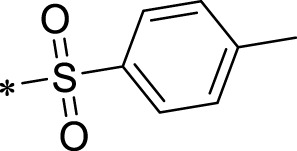	25.49 ± 1.77
N-(3-((3-methylpyridin-2-yl)amino)quinoxalin-2-yl)benzamide			
02	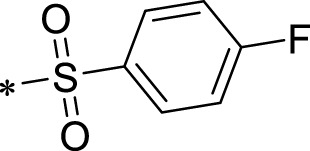	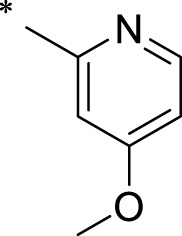	50.12 ± 1.36
N-(3-((furan-2-ylmethyl)amino)quinoxalin-2-yl)-4-methylbenzamide			
02a	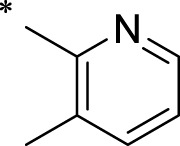	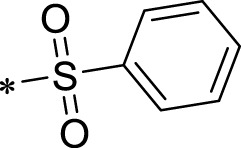	63.46 ± 3.31
N-(3-((furan-2-ylmethyl)amino)quinoxalin-2-yl)-4-methylbenzenesulfonamide			
02b	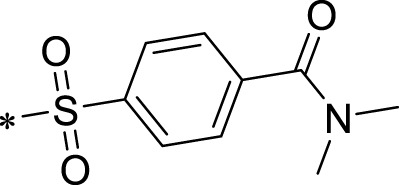	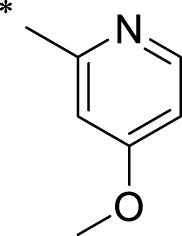	76.66 ± 4.72
N-(3-((furan-2-ylmethyl)amino)quinoxalin-2-yl)benzenesulfonamide			
02c	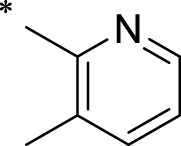	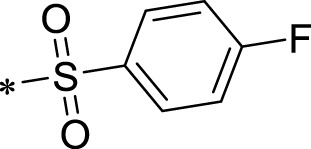	20.30 ± 2.91
4-fluoro-N-(3-((furan-2-ylmethyl)amino)quinoxalin-2-yl)benzenesulfonamide			
02d	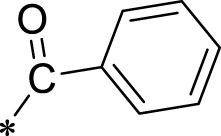	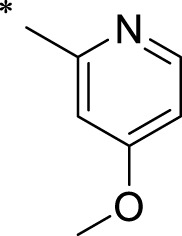	62.96 ± 6.50
N-(3-((furan-2-ylmethyl)amino)quinoxalin-2-yl)benzamide			
03	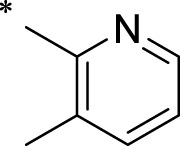	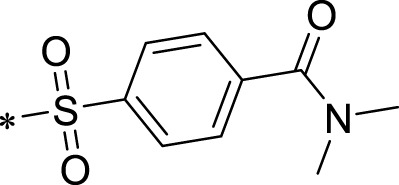	10.88 ± 1.60
N-(3-((4-methoxypyridin-2-yl)amino)quinoxalin-2-yl)-4-methylbenzamide			
03a	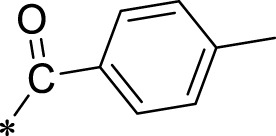	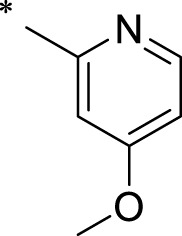	3.52 ± 2.08
N-(3-((4-methoxypyridin-2-yl)amino)quinoxalin-2-yl)-4-methylbenzenesulfonamide			
03b	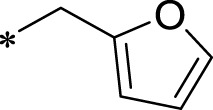	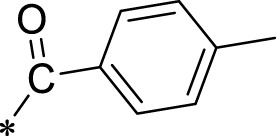	19.73 ± 18.53
N-(3-((4-methoxypyridin-2-yl)amino)quinoxalin-2-yl)benzenesulfonamide			
03c	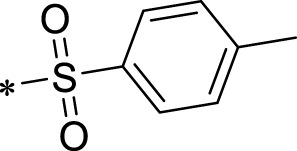	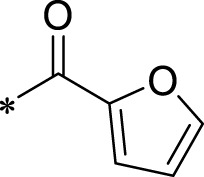	6.97 ± 0.67
4-fluoro-N-(3-((4-methoxypyridin-2-yl)amino)quinoxalin-2-yl)benzenesulfonamide			
03d	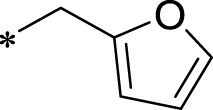	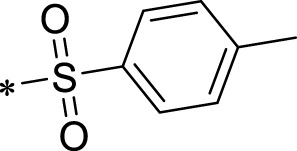	6.96 ± 1.63
4-(N-(3-((4-methoxypyridin-2-yl)amino)quinoxalin-2-yl)sulfamoyl)-N,N-dimethylbenzamide			
04	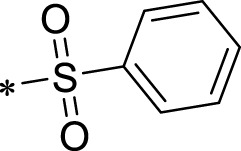	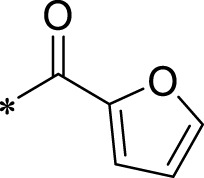	4.09 ± 1.37
N-(3-(4-methylbenzamido)quinoxalin-2-yl)furan-2-carboxamide			
04a	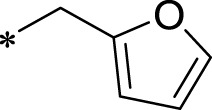	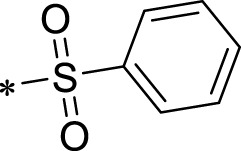	32.06 ± 3.07
N-(3-((4-methylphenyl)sulfonamido)quinoxalin-2-yl)furan-2-carboxamide			
04b	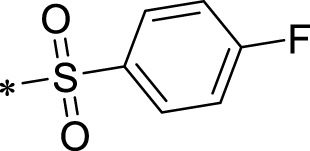	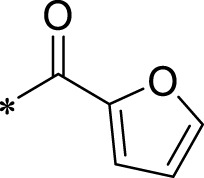	3.65 ± 1.18
N-(3-(phenylsulfonamido)quinoxalin-2-yl)furan-2-carboxamide			

IR, Inhibitory rates (%).

### 3.3 Compound 2b inhibits the direct cleavage of Notch protein in A549 cells

**FIGURE 5:**
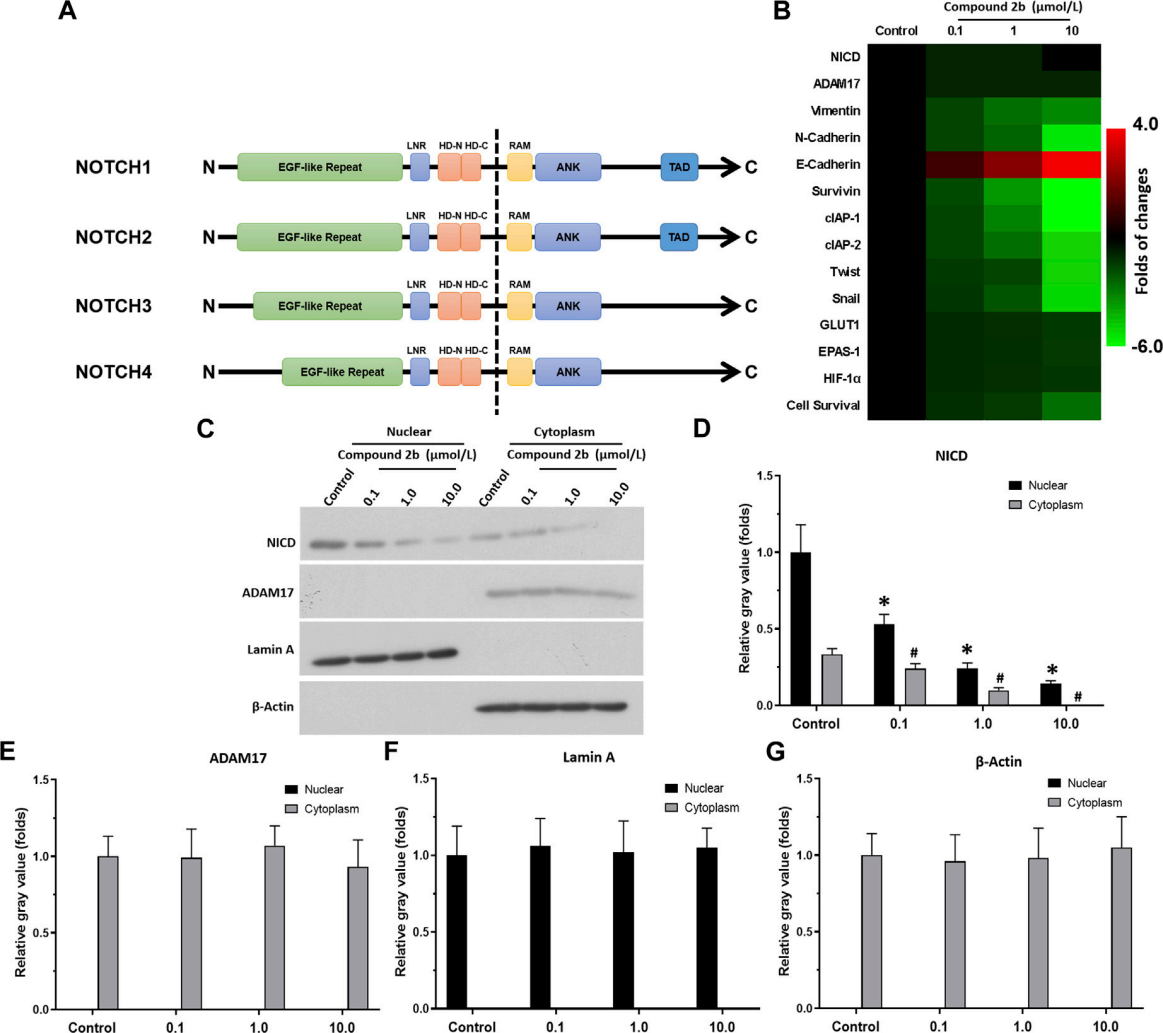
Compound 2b repressed Notch pathway activation **(A)** Schematic diagram of the protein structures of Notch1∼4. The extracellular domains of Notch1∼4 are different, but the intracellular domains are highly conserved. **(B)** A549 cells are treated with the indicated concentrations (0.1 [low dose], 1 [medium dose] or 10 μmol/L [high dose]) of compound **2b** and cells are harvested for RT-qPCR. The expression level of drug-resistance related Notch pathway’s downstream genes in A549 cells treated by the compound **2b** is assessed by RT-qPCR and the results are exhibited as heat-maps according to relative mRNA expression. The reference-scale for heat map is inferred as folds of changes by the control group. Positive folds of changes indicate that the mRNA expression of target genes in cells of compound **2b** treatment group is increased relative to the control group; negative folds of changes indicate that the mRNA expression of target genes in cells of compound **2b** treatment group is decreased relative to the control group. The colour and brightness of the heat map bands visually reflect the expression level of the target genes in each group. **(C–G)** A549 cells are treated with the indicated concentrations (0.1 [low dose], 1 [medium dose] or 10 μmol/L [high dose]) of compound **2b** and subsequently harvested and lysed to obtain subcellular fractions: nuclear and cytoplasmic. The accumulation of NICD (Notch intracellular domain) and the distribution of ADAM17 in the nuclear and cytoplasmic fractions, respectively, is examined using their antibodies. Nuclear skeleton protein Lamin-A and β-Actin are used as indicators of the nuclear and cytoplasmic fraction, respectively, of A549 cells treated by compound **2b**. Results are exhibited as images of western blots **(C)** or quantitatively **(D–G)**. *p < 0.05 for NICD in the nucleus compared to the Control group; #*p* < 0.05 for NICD in the cytoplasm compared to the Control group.

The aforementioned results suggested that compound **2b** could be an ADAM17 inhibitor, which inhibits Notch pathway activation. Figure 5A showed a schematic diagram of Notch1-4, and the activity of Compound 2b is reflected by detecting the expression of drug-resistance related factors in downstream NICD and Notch. RT-qPCR results revealed that the three doses of compound **2b** could significantly inhibit the activation of the Notch pathway; they could inhibit expression of pro-survival/anti-apoptotic and EMT-related factors, all of which downstream genes of Notch pathway (Figure 5B). Treatment of compound **2b** (with primers designed in the conserved NICD region to simultaneously detect the expression levels of the four Notch isoforms and whether compound **2b** affects the expression of the four Notch isoforms) did not affect the expression level of NICD or ADAM17 (Figure 5B). Compound **2b** inhibited the survival of A549 cells in a dose-dependent manner (Figure 5B). Compound **2b** at 10 μmol/L, 1 μmol/L, and 0.1 μmol/L significantly inhibited, weakly inhibited, and had virtually no inhibitory activity, respectively, on A549 cells (Figure 5B). Among these three doses, 1 μmol/L did not have significant cytotoxicity and it could significantly inhibit Notch pathway activation (Figure 5B); therefore, this dose was selected for further experiments.

The four Notch protein isoforms (NOTCH1-4) have similar functions and mutual compensatory effects. It is evident that while the extracellular domain of the four Notch isoforms significantly differs, the intracellular segments are highly conserved and largely uniform. Therefore, to ascertain the effect and specificity of compound **2b** on ADAM17/Notch pathway, the accumulation of NICD (Notch intracellular domain) of Notch proteins in the nuclear sub-fraction of NSCLC cells was examined. As exhibited in Figures 5C–G, treatment with compound **2b** did not modulate ADAM17 expression in the cytoplasmic fraction of A549 cells, whereas it reduced NICD accumulation in the nuclear fraction of A549 cells in a dose-dependent manner (Figures 5C–G). These data suggested that compound **2b** inhibited ADAM17’s activation to inhibit the direct cleavage of Notch protein, which in turn inhibited Notch pathway activation.

### 3.4 Compound 2b enhances the sensitivity of NSCLC cells to antitumour drugs

The aforementioned results suggested that compound **2b** inhibited the ADAM17/Notch pathway activation, which mediates the resistance of cancer cells to antitumour drugs. To further examine the effect of compound **2b** on the Notch pathway, the MTT assay was performed to ascertain if compound **2b** inhibited the survival of A549 cells; furthermore, the sensitivity of NSCLC cells to antitumour drugs, i.e., whether cells were affected by compound **2b,** were ascertained. As presented in the Tables, treatment with antitumour drugs (erlotinib, gefitinib, afatinib, osimertinib, anlotinib, gemcitabine, epirubicin, docetaxel, and oxaliplatin) inhibited the survival of A549 cells in a dose-dependent manner, and the *IC*_*50*_ values of these drugs are listed in Table 6. Pre-treatment with compound **2b** enhanced the sensitivity of A549 cells to antitumor drugs and the *IC*_*50*_ values of these antitumour drugs decreased. To confirm the specificity of compound **2b**, an expression vector of NICD (expression vector for the full sequence of NICD segment of Notch1 protein) was transfected into A549 cells. NICD overexpression nearly blocked the effect of compound **2b** on these antitumour drugs (Table 6). Similar results were obtained from the cell lines H460 and H520 (Table 6). Therefore, compound **2b** enhanced the sensitivity of NSCLC cells to antitumour drugs.

**TABLE 6:** Treatment of compound 2b enhances the sensitivity of NSCLC cells to antitumor drugs.

Cell lines	Antitumor drugs	Control	Compound 2b	Compound 2b + NICD
		*IC*_*50*_ values (μmol/L)		
A549	Erlotinib	0.73 ± 0.36	0.46 ± 0.03	0.80 ± 0.33
	Gefitinib	0.99 ± 0.58	0.25 ± 0.09	1.06 ± 0.57
	Afatinib	0.85 ± 0.24	0.20 ± 0.06	0.97 ± 0.55
	Osimertinib	0.63 ± 0.08	0.35 ± 0.10	0.74 ± 0.32
	Anlotinib	0.50 ± 0.16	0.22 ± 0.13	0.57 ± 0.40
	Oxaliplatin	0.35 ± 0.07	0.05 ± 0.01	0.29 ± 0.08
	Docetaxel	0.05 ± 0.01	0.01 ± 0.00	0.11 ± 0.03
	Epirubicin	0.22 ± 0.05	0.10 ± 0.04	0.20 ± 0.05
	Gemcitabine	0.23 ± 0.02	0.07 ± 0.02	0.36 ± 0.30
H460	Erlotinib	0.85 ± 0.53	0.24 ± 0.07	0.71 ± 0.45
	Gefitinib	1.01 ± 0.28	0.60 ± 0.05	0.83 ± 0.25
	Afatinib	0.91 ± 0.47	0.36 ± 0.24	0.81 ± 0.37
	Osimertinib	0.73 ± 0.34	0.45 ± 0.13	0.75 ± 0.14
	Anlotinib	0.68 ± 0.26	0.32 ± 0.19	0.76 ± 0.33
	Oxaliplatin	0.40 ± 0.12	0.11 ± 0.04	0.39 ± 0.04
	Docetaxel	0.11 ± 0.03	0.03 ± 0.01	0.18 ± 0.05
	Epirubicin	0.51 ± 0.13	0.18 ± 0.00	0.76 ± 0.42
	Gemcitabine	0.33 ± 0.07	0.06 ± 0.04	0.31 ± 0.16
H520	Erlotinib	0.78 ± 0.65	0.20 ± 0.09	0.82 ± 0.26
	Gefitinib	0.63 ± 0.45	0.24 ± 0.03	0.74 ± 0.80
	Afatinib	0.80 ± 0.34	0.43 ± 0.17	0.75 ± 0.25
	Osimertinib	0.61 ± 0.38	0.35 ± 0.09	0.92 ± 0.81
	Anlotinib	0.68 ± 0.49	0.26 ± 0.11	0.64 ± 0.33
	Oxaliplatin	0.48 ± 0.21	0.22 ± 0.12	0.40 ± 0.25
	Docetaxel	0.12 ± 0.06	0.03 ± 0.00	0.21 ± 0.05
	Epirubicin	0.51 ± 0.30	0.17 ± 0.08	0.62 ± 0.17
	Gemcitabine	0.24 ± 0.08	0.15 ± 0.12	0.30 ± 0.06

### 3.5 Compound 2b enhances the sensitivity of A549 cells to anlotinib *in vivo*

**FIGURE 6:**
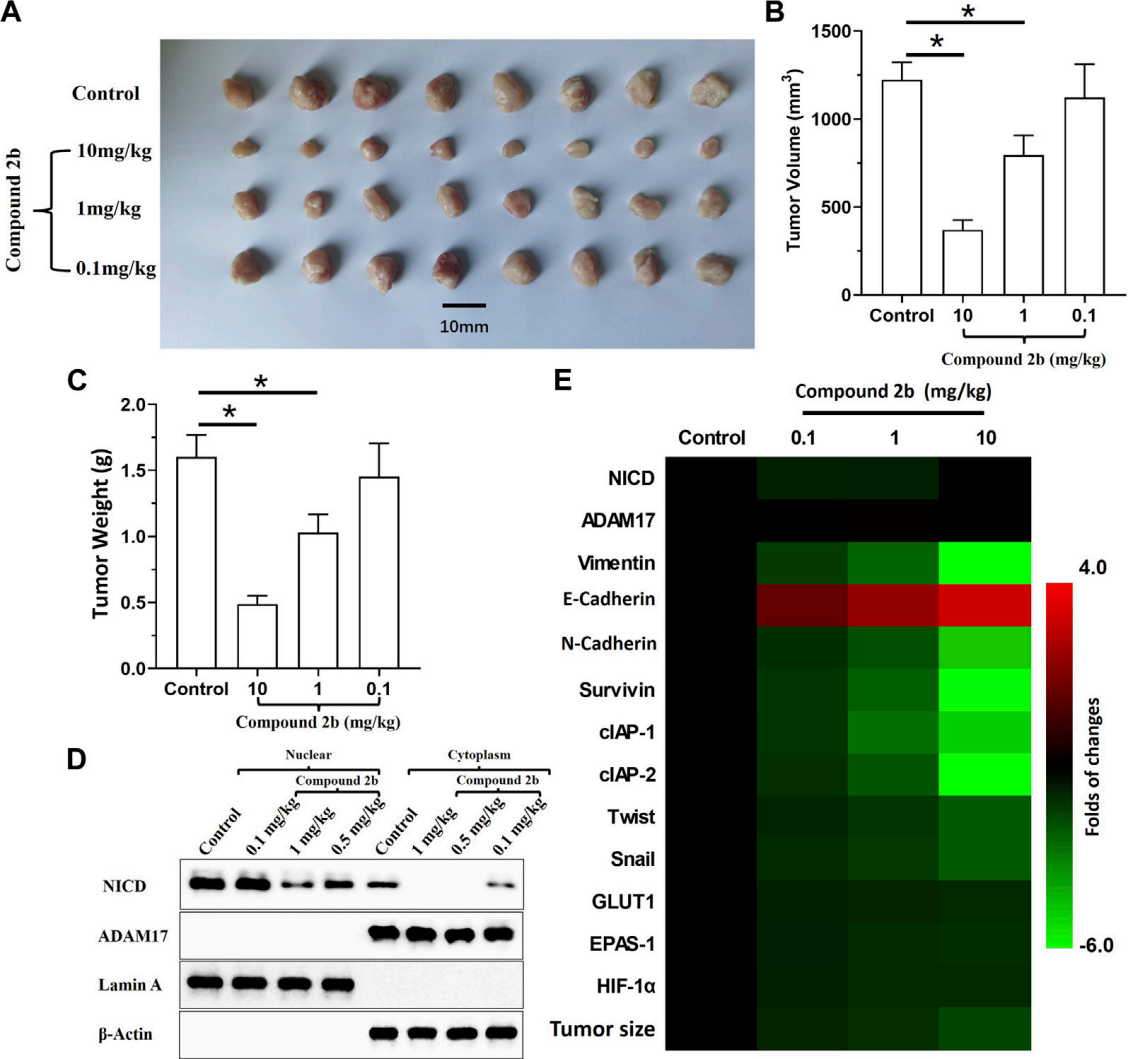
Inhibitory effect of compound **2b** on the subcutaneous growth of A549 cells and expression of the Notch pathway-related factors in tumour tissues. Nude mice are subcutaneously injected (medial inguinal area adjacent to the femoral vein) with A549 cells (5 × 106 per animal). The mice are orally administered the indicated concentrations (10 [high dose], 1 [medium dose], and 0.1 [low dose] mg/kg) of compound **2b**. The tumours are collected, imaging is performed and the tumour volumes and weight are assessed. The expression of Notch pathway-related factors in the subcutaneous tumour tissues are examined by RT-qPCR. Cells from the subcutaneous tumour tissue were obtained by grinding using a pre-sterilised 200 mesh steel sieve (Eight tumour tissues per group were mixed). The cells were lysed to obtain subcellular fractions: nuclear and cytoplasmic. The accumulation of NICD (Notch intracellular domain) and the distribution of ADAM17 in the nuclear and cytoplasmic fractions, respectively, was examined using their antibodies. Nuclear skeleton protein Lamin-A and β-Actin were used as indicators of the nuclear and cytoplasmic fraction, respectively. Results are exhibited as images of western blots Results are exhibited as images of tumour tissues **(A)**, quantitative data **(B, C)**, the images of Western blot **(D)** and heat-maps of Notch-related factors in tumours assessed *via* RT-qPCR (E). *p < 0.05.

An *in vivo* tumour model was used to further examine if compound **2b** enhanced the sensitivity of NSCLC cells to antitumour drugs. A549 cells could form subcutaneous tumour tissues in nude mice (Figure 6). Treatment with compound **2b** inhibited the subcutaneous growth of A549 cells in a dose-dependent manner (Figures 6A–C). Three doses of compound **2b** (10, 1, or 0.1 mg/kg) could significantly inhibit the activity of the Notch pathway (i.e., the accumulation of NICD in nuclear sub-faction of cells but not affect the expression of ADAM17 (Figure 6D); or the expression of Notch pathway’s downstream genes in the subcutaneous tumour tissues (Figure 6E)]; they could significantly inhibit expression of drug resistance-related factors downstream of the Notch pathway without affecting expression of NICD or ADAM17 (Figure 6 D and E). Compound **2b** (10 mg/kg) significantly inhibited the subcutaneous growth of A549 cells (Figures 6A–C), whereas 0.1 mg/kg or 1 mg/kg had weak inhibitory activity on A549 cells compared with the 10 mg/kg dose (Figure 6). Therefore, compound **2b** at 1 mg/kg was selected for the further experiments.

**FIGURE 7:**
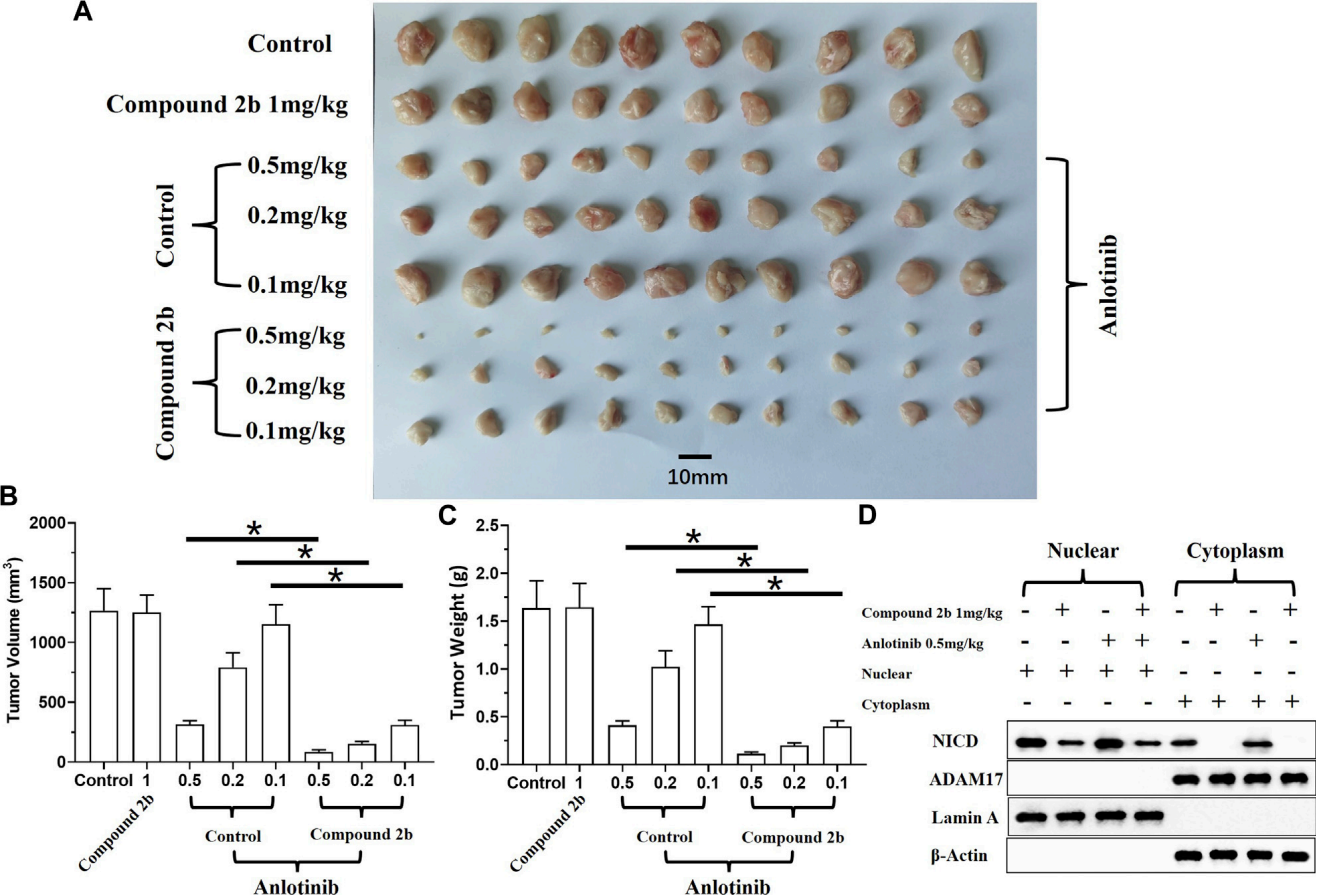
Treatment with compound **2b** enhances the sensitivity of A549 cells to anlotinib in a subcutaneous tumour model. Nude mice are subcutaneously injected (medial inguinal area adjacent to the femoral vein) with A549 cells (5×10^6^ per animal). The mice are orally administered compound **2b** (1 mg/kg dose) alone, anlotinib (0.5 mg/kg [high dose] or 0.2 mg/kg [medium dose], or 0.1 mg/kg [low dose]) alone, or anlotinib (0.5 mg/kg [high dose], 0.2 mg/kg [medium dose], or 0.1 mg/kg [low dose]) + compound **2b** (1 mg/kg). The tumours are collected, imaging is performed, and tumour volumes and weight are assessed. Cells from the subcutaneous tumour tissue were obtained by grinding using a pre-sterilised 200 mesh steel sieve (Eight tumour tissues per group were mixed) (Tumour tissue of the control group, 1 mg/kg compound 2b group, 1 mg/kg compound 2b + 0.5 mg/kg Anlotinib group and 0.5 mg/kg Anlotinib group were ground to extract single cell suspensions). The cells were lysed to obtain subcellular fractions: nuclear and cytoplasmic. The accumulation of NICD (Notch intracellular domain) and the distribution of ADAM17 in the nuclear and cytoplasmic fractions, respectively, was examined using their antibodies. Nuclear skeleton protein Lamin-A and β-Actin were used as indicators of the nuclear and cytoplasmic fraction, respectively. Results are exhibited as images of tumour tissues **(A)**, quantitative data **(B, C)**, and images of Western blot **(D)**. *p < 0.05.

The effect of compound **2b** combined with a TKI (anlotinib) on A549 cells was examined in both the subcutaneous and intra-lung tumour models. The results revealed that anlotinib could inhibit the subcutaneous growth of A549 cells in the nude mice models in a dose-dependent manner (Figure 7). The antitumor activity of anlotinib was significantly enhanced if combined with compound **2b** 1 mg/kg (Figure 7). To confirm the effect of **compound 2b**, the cells were separated from the subcutaneous tumor tissues for cellular sub-fraction assays. As shown in Figure 7D, neither treatment of compound 2b nor Anlotinib affect the expression level of ADAM17 in A549 cells. Moreover, treatment of 1 mg/kg dose of compound **2b** but not 0.5 mg/kg Anlotinib inhibited the accumulation of NICD in the nuclear sub-faction of cells (Figure 7D).

**FIGURE 8:**
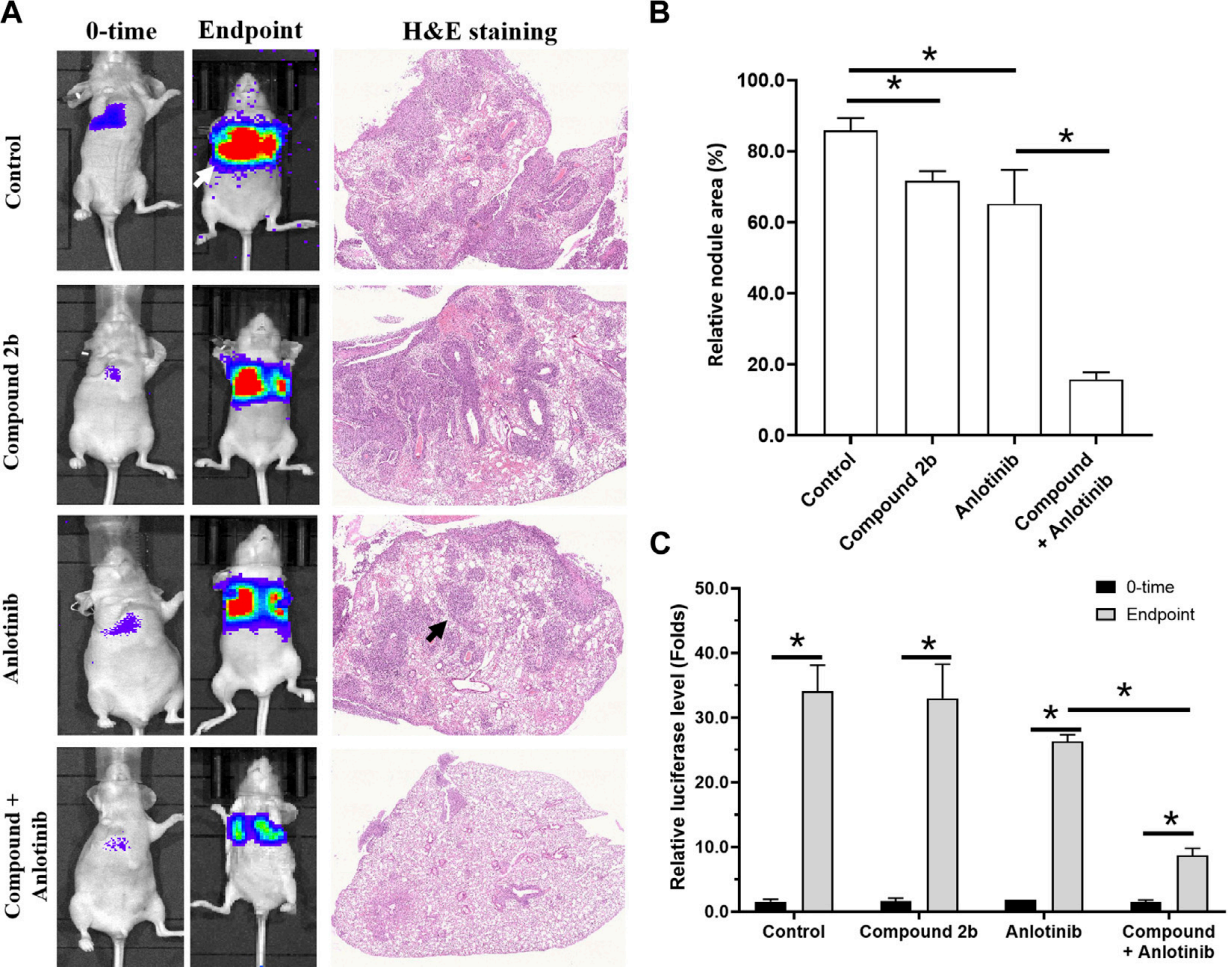
Treatment with compound **2b** enhances the sensitivity of A549 cells to anlotinib in an intra-lung tumour model. A549 (2×10^5^ per animal) cells labelled by EGFP-luciferase are injected *via* the tail vein of nude mice to form lung tumours. Subsequently, the mice are imaged with a luciferase-based imaging system as the 0-time point. The mice are orally administered compound **2b** (1 mg/kg dose) alone, anlotinib (0.2 mg/kg) alone, or anlotinib (0.2 mg/kg) + compound **2b** (1 mg/kg). After drug treatment (oral administration), the mice are imaged with the luciferase-based imaging system as the end-time point. After *in vivo* imaging, the nude mice are euthanised and the lungs are harvested for pathological staining (H & E staining). Results are exhibited as *in vivo* images based on luciferase or H & E staining **(A)** and quantitative data **(B, C)**. For images of the animals, the lung-region luciferase signal is directly quantified using a live imaging workstation; for pathological staining results, the total area of lung tissue and lesions are calculated separately using the ImageJ software, and the lesions are quantified based on the total area of lung tissue. *p < 0.05. The white arrow indicates the luciferase image in the lungs of the nude mice; the black arrow indicates the lesions formed by A549 cells in the pathological staining results/images.

Next, in the intra-lung tumour model, A549 cells formed nodules/lesions in the lungs of the nude mice, after a tail-vein injection of a suspension of A549 cells (Figure 8). These nodules/lesions in the nude mice’s lungs could be detected by a luciferase-based, small-animal *in vivo* imaging system and pathological staining (Figure 8). The anti-tumour activity of compound **2b** (1 mg/kg) alone and anlotinib (0.2 mg/kg) alone was limited (this dose of compound **2b** did not have significant cytotoxicity/the cytotoxicity of this dose of compound **2b** was weak); however, a combination of these two agents exhibited extremely potent anti-tumour activity (Figure 8). Therefore, compound **2b** enhances the sensitivity of A549 cells to anlotinib *in vivo*.

### 3.6 Conformation of compound 2b interacts with ADAM17

**FIGURE 9:**
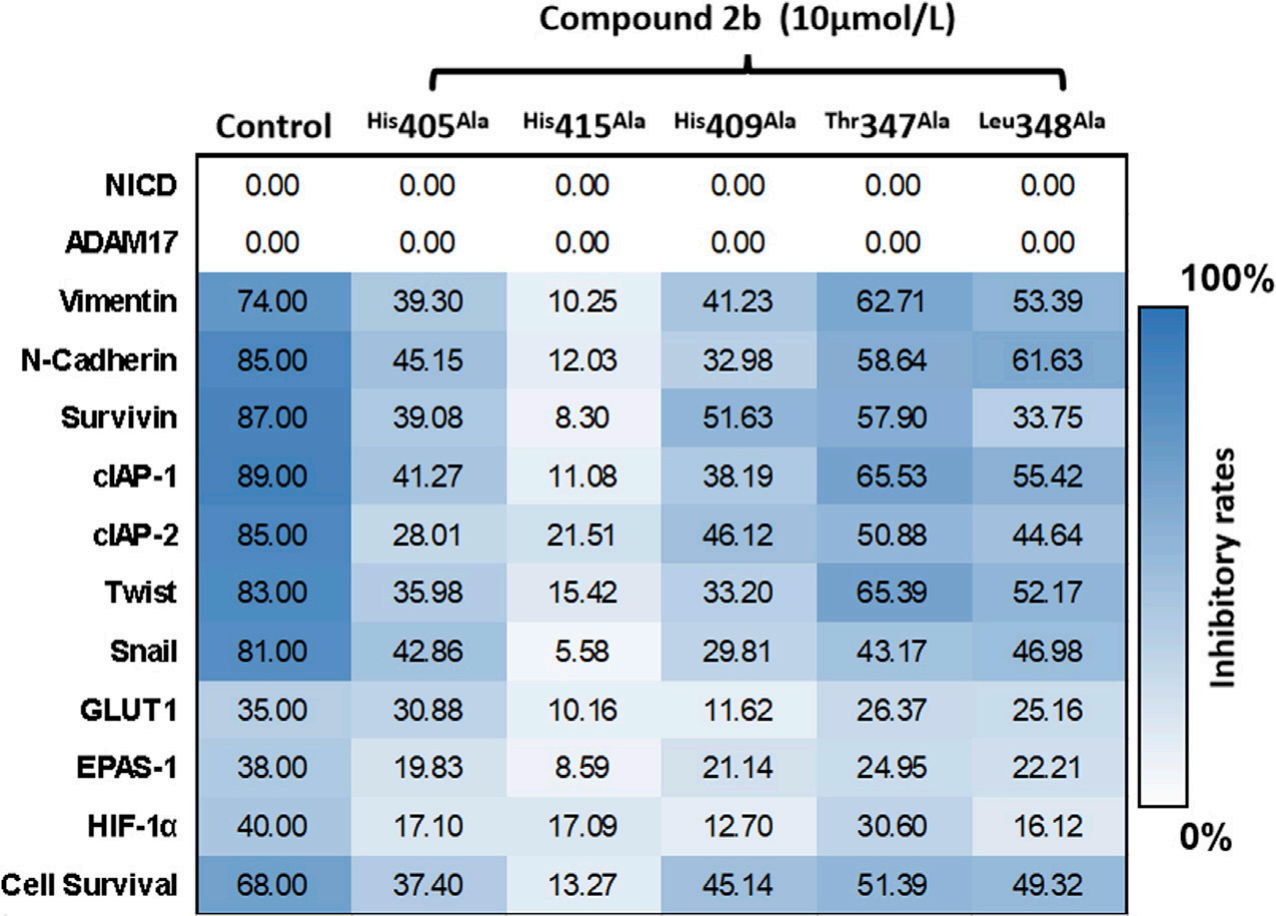
Specificity of compound **2b** on ADAM17 confirmed by a point-mutation method. ADAM17 is prepared with point mutations (His 405, His 415, His 409, Thr 347, or Leu 348 are replaced by alanine). A549 cells are transfected with these point-mutation vectors and treated with compound **2b** 10 μmol/L. Subsequently, the cells are harvested for qPCR. The mRNA expression of Notch pathway-related factors is measured by the qPCR in the blank group (A549 cells do not transfect vectors or treated with compound **2b**), control group (A549 is transfected with empty vectors and treated with 10 μmol/L dose of compound **2b**), or ADAM17 mutated group (A549 is transfected with ADAM17 point mutation and treated with compound **2b** 10 μmol/L). The inhibitory rates of compound **2b** at each group is measured as follows (mRNA level of target gene in blank group - mRNA level of target gene in control group)/(mRNA level of target gene in blank group) × 100%; or (mRNA level of target gene in blank group - mRNA level of target gene in ADAM17 mutated group)/(mRNA level of target gene in blank group) × 100%. Results are exhibited as heat-maps according to these inhibitory rates.

Herein, we have summarized the possible conformational relationships between the resulting small molecule inhibitors and ADAM17 interactions in terms of molecular mechanisms. According to the molecular-docking results of the lead compound/Compound **2a** (Figure 4A) or compound **2b** (Supplementary Figure S1) and ADAM17, the key amino-acid disability His 405, His 415, His 409, Thr 347, or Leu 348, in ADAM17 that interacts with compound **2b** was replaced by alanine (Figure 9). At the same time, Compound **2b** has one less methyl group than leading compound/Compound **2a**, which may have reduced the site resistance of the compound, allowing for a tighter π-π conjugation with HIS405 (Figure 4A; Supplementary Figure S1). From the activity of Compounds **3** to Compound **3d**, it is also clear that the larger the substituent on the benzene ring in the R1 group, the more it reduces the activity of the compound instead (Table 4). Furthermore, compounds containing Sulfonyl benzene clearly show better activity than those containing acyl benzene, probably because Sulfonyl benzene contains two hydrogen bond acceptors and is better angled for hydrogen bonding with THR347. For the R2 groups, compounds containing hydrogen bond acceptors are preferred, with the furan group is relatively best (Figure 4; Supplementary Figure S1; Table 4).

Subsequently, the expression vector of the point mutation of ADAM17 with these mutated key amino-acids was constructed to transfect A549 cells, which in turn were treated with a 10 μmol/L dose of compound **2b** (Figure 9). Since these amino-acid residues were mutated, the inhibitory effect of compound **2b** on ADAM17 was weakened; this was reflected in a significant decrease in inhibitory rates of compound **2b** on the downstream-related factors of the Notch pathway (Figure 9). Comparison of His 405, His 415, His 409, Thr 347, or Leu 348 revealed that the effect of a point mutation on the first group of amino-acid residues (His 405, His 415, or His 409) on compound **2b** exceeded that of the second group of amino-acid residues (Thr 347 or Leu 348). Furthermore, among the first group of amino-acid residues (His 405, His 415, or His 409), replacing His 415 by alanine had the largest effect on compound **2b** in reducing the inhibitory activity of compound **2b** on the Notch pathway (Figure 9). Therefore, His 415 may be the most important amino-acid residue in the interaction between ADAM17 and compound **2b.**

**FIGURE 10:**
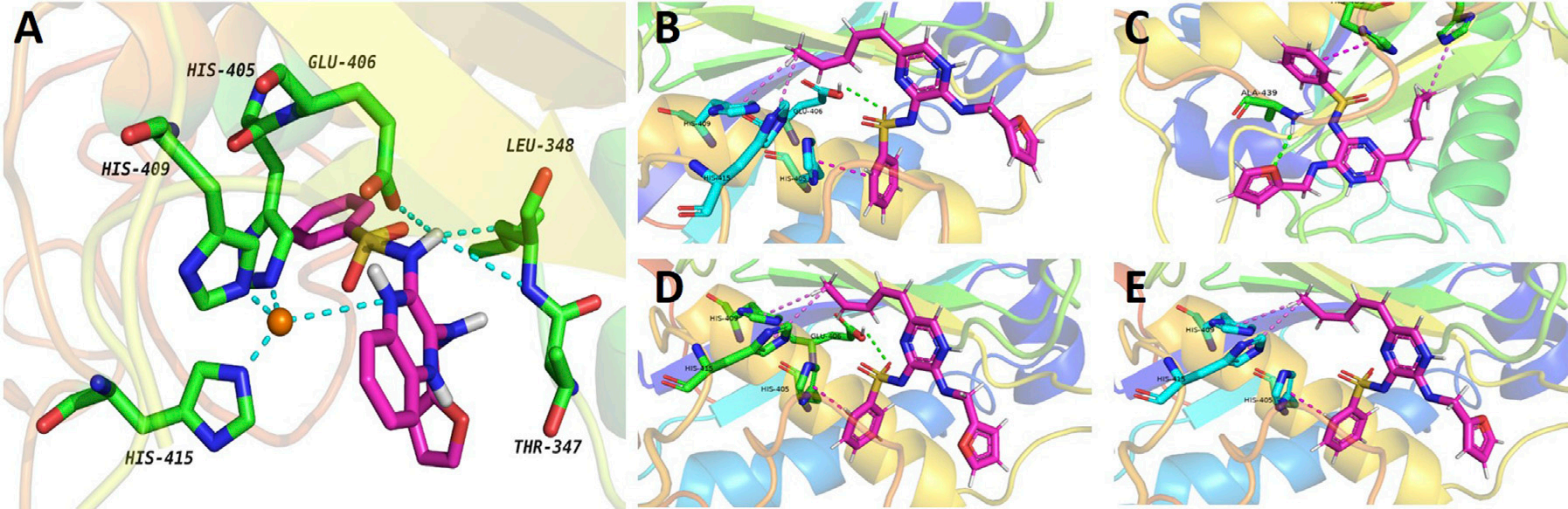
Potential interaction of ADAM17-based small-molecule inhibitor compound 2b (the preferred compound) with ADAMs *via* molecular-docking. Molecular docking of compounds to proteins. Virtual docking of compounds to protein targets was performed using Autodock 4.2.6 and the results were visualised and analysed by PyMOL 2.2.0 software. The results were shown as the images of molecular docking between compound 2b with ADAM17 **(A)**, ADAM33 **(B)**, ADAM22 **(C)**, ADAM10 **(D)** and ADAMTS-4 **(E)**.

Furthermore, the potentially selection of ADAMs by compound 2b was examined. The results showed that the docking binding energy of ADAM17 to compound **2b** was −9.1 kcal/mol, and the binding ability was better than other targets (ADAM33, ADAM22, ADAM10, or ADAMTS-4), with compound **2b** preferring to bind to ADAM17 (Table 7). The docking binding energy of ADAM17, ADAM33, ADAM22, ADAM10, or ADAMTS-4 to compound **2b** was shown as Table 7. The binding modes were shown as Figure 10: the binding pattern of compound **2b** to ADAM17 (Figure 10A) is different from that of several other ADAMs (Figures 10B–E). This further reveals that compound 2b targets selectively on ADAMs.

**TABLE 7:** The docking binding energy of ADAMs to compound 2b.

Proteins	The docking binding energy (kcal/mol)
ADAM17	−9.1
ADAM33	−8.3
ADAM22	−7.3
ADAM10	−7.6
ADAMTS-4	−7.0

### 3.7 Comparative of the activity of compound 2b and other small molecular inhibitors of ADAM17

To further examine the activation of compound **2b**, the comparative of the activity of compound 2b and other small molecule inhibitors of ADAM17 was performed. There are three existing small molecule inhibitors of ADAM17: pratastat, terfenadine and ZLDI-8. Overall, the activity of compound **2b** is superior to these three existing compounds. This was reflected in the fact that the binding energy of compound **2b** to ADAM17 is lower than that of pratastat, terfenadine and ZLDI-8 (Table 8), and that the *IC*_*50*_ values of compound **2b** for inhibition of ADAM17 activity (inhibition of Survivin, a Notch downstream resistance factor) are lower than those of pratastat, terfenadine and ZLDI-8 both in A549, H460 or H520 cells (Table 9). The *IC*_*50*_ values of compound **2b** to inhibit the survival of NSCLC cells was also much lower compared with the pratastat, terfenadine and ZLDI-8 (Table 10).

**TABLE 8:** The docking binding energy between the existing inhibitors of ADAM17 with ADAM17.

Agents	The docking binding energy (kcal/mol)
compound 2b	−9.1
pratastat	−8.7
terfenadine	−8.2
ZLDI-8	−8.4

**TABLE 9:** The *IC*_*50*_ values of the existing inhibitors of ADAM17 on the Notch pathway in NSCLC cells.

Agents	A549	H460	H520
	*IC*_*50*_ on survivin		
compound 2b	1.47 ± 0.71	1.65 ± 0.42	1.26 ± 0.31
pratastat	1.95 ± 0.18	1.77 ± 0.40	1.55 ± 0.67
terfenadine	2.61 ± 0.44	1.81 ± 0.23	1.86 ± 0.66
ZLDI-8	2.02 ± 0.93	1.93 ± 0.20	1.83 ± 0.35

The NSCLC, cells were cultured and treated for the indicated concentrations of the existing inhibitors (compound 2b, pratastat, terfenadine, ZLDI-8) of ADAM17. The cells were harvested for the qPCR, to examine the expression of Survivin, which is a typical downstream gene of Notch pathway. The inhibition rate of Survivin expression by different doses of the drug on NSCLC, cells was used to calculate the *IC*_*50*_ values and ultimately reflect the inhibitory activity of the drug on the Notch pathway.

**TABLE 10:** The *IC*_*50*_ values of compound 2b, pratastat, terfenadine or ZLDI-8 on the survival of NSCLC cells.

Agents	A549	H460	H520
	*IC*_*50*_ values (μmol/L)		
compound 2b	24.49 ± 7.18	22.69 ± 3.88	26.74 ± 5.41
pratastat	>30	>30	29.66 ± 7.03
terfenadine	>30	25.76 ± 8.10	∼30
ZLDI-8	29.45 ± 4.67	28.65 ± 5.91	>30

The NSCLC, cells were cultured and treated for the indicated concentrations of the existing inhibitors (compound 2b, pratastat, terfenadine, ZLDI-8) of ADAM17. The cells were harvested for the MTT, assays. The inhibition rate of cell’s survival by different doses of the drug was used to calculate the *IC*_*50*_ values.

Next, the effect of compound **2b**, pratastat, terfenadine and ZLDI-8 on the antitumor effects of Gefitinib on A549 cells was examined. As shown in Table 11, all four drugs were able to upregulate the sensitivity of A549 cells to Gefitinib, as evidenced by the ability of compound **2b**, pratastat, terfenadine and ZLDI-8 to significantly downregulate the *IC*_*50*_ values of Gefitinib on A549. In contrast, the sensitisation effect of compound **2b** on Gefitinib in A549 cells was significantly stronger than that of pratastat, terfenadine and ZLDI-8, with sensitisation multipliers of 4.91, 1.86, 2.70 and 3.27 for these drugs respectively (Table 11). These results revealed the advantage of compound 2b compared with the existing inhibitor of AMAD17.

**TABLE 11:** The existing inhibitor of ADAM17 enhanced the sensitivity of A549 cells to Gefitinib.

Agents	IC50 values (μmol/L) of gefitinib	Sensitization folds
control	1.08 ± 0.37	—
compound 2b	0.22 ± 0.06	4.91
pratastat	0.58 ± 0.14	1.86
terfenadine	0.40 ± 0.20	2.70
ZLDI-8	0.33 ± 0.04	3.27

A549 cells were treated with 1 μmol/L dose of compound 2b with the indicated concentrations of Gefitinib. The *IC*_*50*_ values of Gefitinib on A549 cells combined with the existing inhibitor of ADAM17 was calculated. Sensitization folds were calculated as (the *IC*_*50*_ value of Gefitinib)/(the IC50 value of Gefitinib combined with the existing inhibitor of ADAM17).

### 3.8 Synergy index assay of compound 2b and Gefitinib combinational treatment for NSCLC cells

**FIGURE 11:**
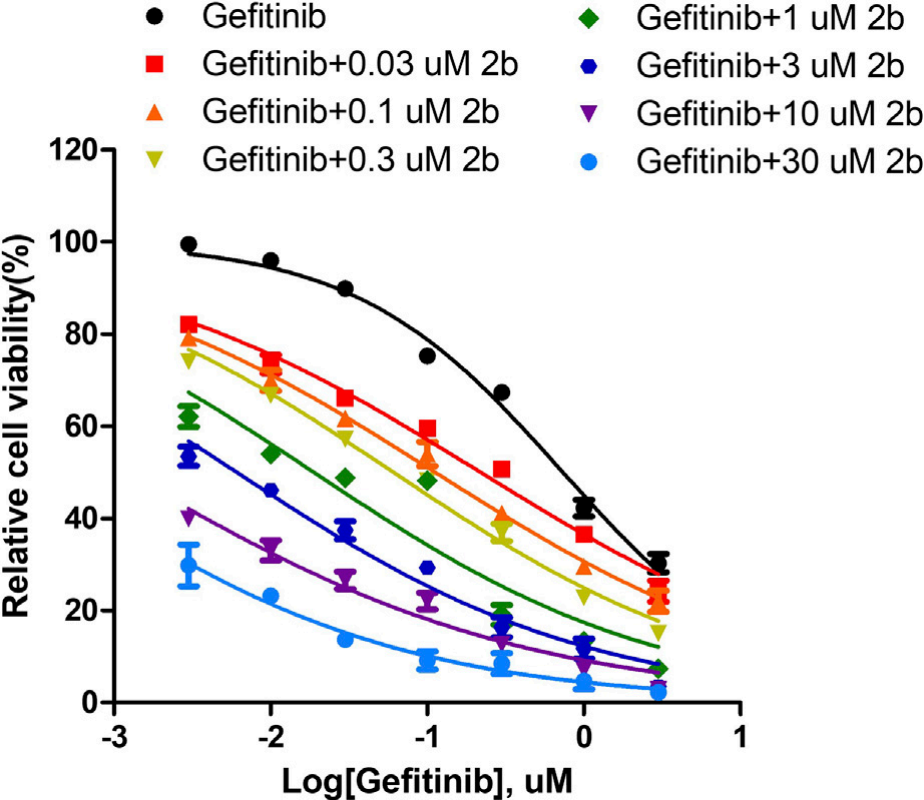
The Sensitization to Gefitinib by compound 2b in A549 cells. A549 cells were obtained in culture, and the inhibition rate of each group was measured by MTT assay using 30, 10, 3, 1, 0.3, 0.1 and 0.03 μmol/L doses of compound 2b in combination with Gefitinib (3, 1, 0.3, 0.1, 0.03, 0.01 and 0.003 μmol/L), respectively. The effect of different doses of compound 2b on the IC50 value of Gefitinib was finally determined.

The results described above are for a single dose of compound **2b** in combination with multiple doses of Gefitinib, but for a more comprehensive analysis we finally tested a series of doses of compound **2b** in combination with a series of doses of Gefitinib (Figure 11). The results showed that the *IC*_*50*_ values of Gefitinib were significantly lower in A549 cells when combined with compound **2b** compared to the control group (Table 12). At this point, the higher the dose of compound **2b** in combination, the lower the *IC*_*50*_ value of Gefitinib. For example, the *IC*_*50*_ value of Gefitinib in the control group was 0.74 ± 0.22 μmol/L, the *IC*_*50*_ value of Gefitinib in the 1 μmol/L compound **2b** group was 0.18 ± 0.09 μmol/L, and the *IC*_*50*_ value of Gefitinib in the 3 μmol/L compound **2b** group was 0.02 ± 0.01 μmol/L. Out of the *IC*_*50*_ values, the synergy index was also calculated: dual treatment of compound **2b** with Gefitinib showed higher combinational inhibitory activation compared to the single-treatment of compound 2b or Gefitinib, as these two compounds synergy Index <1 (Table 13), which reflected the potential synergistic effect between compound 2b with Gefitinib on A549 cells. These results further confirmed the effect of compound 2b on Gefitinib in NSCLC cells.

**TABLE 12:** The *IC*_*50*_ values of Gefitinib combined with the different concentration of compound 2b on A549 cells.

Compounds 2b concentration (μmol/L)	IC50 values of gefitinib (μmol/L)
-	0.74 ± 0.22
0.03	0.51 ± 0.10
0.1	0.45 ± 0.08
0.3	0.33 ± 0.14
1	0.18 ± 0.09
3	0.02 ± 0.00
10	<0.003 μmol/L
30	<0.003 μmol/L

Different doses of Gefitinib were combined with different doses of compound 2b to calculate the *IC*_*50*_ values for different groups of Gefitinib.

**TABLE 13:** Synergistic index of Gefitinib in combination with Compound 2b in A549 cells.

Groups	A549
Fa = 0.50	0.055
Fa = 0.75	0.044
Fa = 0.90	0.037

## 4 Discussion

The development and progression of NSCLC is a multi-step process and the aberrant protein expression induced by genetic changes could be considered potential targets for NSCLC treatment (Baumgart et al., 2010). EGFR or echinoderm microtubule-associated protein-like 4 and anaplastic lymphoma kinase are the most common targets for NSCLC (especially LUAD-related treatment) (Baumgart et al., 2010). Recently, other factors (LKB1/STK11, NF1, CDKN2A, SMARCA4, KEAP1, KRAS, c-MET, ROS1, RET PTEN, KEAP1, MLL2, HLA-A, NFE2L2, RB1, and NOTCH1) have been identified in NSCLC and could be considered novel targets for treatment (Sharma et al., 2016; Solassol et al., 2019). We have focused Notch pathway inhibition by targeting ADAM17. The difference between the four Notch (NOTCH1–4) receptors is mainly in the extracellular segment (the intracellular segment is highly conserved), which features in canonical signalling and several types of human malignancies (Herbst et al., 2018; Duma et al., 2019). The core function of the Notch pathway is to regulate cell-fate decisions, such as inducing expression of pro-survival and anti-apoptotic factors in cells and responding to cell injury/stress factors (Kawaguchi and Kaneko, 2021; Shen and Reedijk, 2021). Notch protein can be activated only by the cleavage of ADAM17 to release NICD. NICD accumulation in nuclei mediates the downstream factors of the Notch pathway. NICD activates the CBF-1/Su(H)/LAG1 transcription factor or mastermind-like transcriptional co-activator genes (Kang et al., 2013).

Using molecular docking and virtual screening, we obtained a series of small-molecule compounds that act on ADAM17. Among these, compound **2b** had a relatively high activity, and was considered the preferred compound. Compound **2b** could inhibit the Notch pathway activity and increase the sensitivity of NSCLC cells to various antitumour drugs. These drugs included TKIs acting on NSCLC cells and some commonly used chemotherapy agents. ICIs were not used in our study; however, the Notch pathway has reportedly been associated with the therapeutic effect of ICIs (Jia et al., 2016; Sprinzak and Blacklow, 2021). Cell lysis—to obtain nuclear and cytoplasmic fractions—and Western blotting were used to detect NICD accumulation in nuclei. Treatment with compound **2b** could reduce and directly inhibit NICD distribution in A549-cell nuclei, which indicated the effect of compound **2b** on ADAM17.

Achieving inhibition of spliced Notch protein by targeting ADAM17 is a rational approach; however, four other strategies to inhibit ADAM17 activation are available. First, development of specific hydroxamate inhibitors for ADAM17. Second, tissue inhibitors of metalloproteinases (TIMPs), especially TIMP-3, could function as natural ADAM17 inhibitors (Long et al., 2021). Third, by using phage-display strategies, neutralizing antibodies against human ADAM17 could be developed as selective ADAM17 inhibitors (Li et al., 2021). Fourth, the recombinant expressed pro-domain of ADAM17 could function as an ADAM17 inhibitor (Navarro et al., 2022). Zhao et al. have made considerable progress in developing small-molecule inhibitors of ADAM17 (Geribaldi-Doldán et al., 2018; Lu et al., 2020; Jia et al., 2021b; Hedges et al., 2022). The cardiotoxic antihistamine drug terfenadine was found to inhibit ADAM17 and exert auxiliary anti-tumour activity through the “old drug, new use" principle. Subsequent studies revealed terfenadine and ZLDI-8 to be promising small-molecule compounds (Geribaldi-Doldán et al., 2018; Lu et al., 2019; Lu et al., 2020; Jia et al., 2021b; Calligaris et al., 2021; Hedges et al., 2022). Compounds with similar structures may be more active than terfenadine (Li et al., 2021).

Virtual screening, molecular docking, and optimization of compound structures were used to design, synthesize, and obtain a preferred small-molecule inhibitor (compound **2b**) with novel structural characteristics. Compared with another compound (**2a**) with high activity, compound **2b** has one fewer methyl group than compound **2a**. This feature may reduce the steric hindrance of compound **2b** and tighten the π–π conjugation between His 415 and His 405. Our point-mutation experiments confirmed this hypothesis; after mutation of His 415, compound **2b** lost its effect upon ADAM17. Further, a larger the substituent on the benzene ring in the R1 group resulted in a lower activity of the compounds. In addition, compounds containing a sulfonyl benzene group demonstrated a significantly higher activity than those containing an acyl benzene group. This effect may be because the sulfonyl benzene group contains two hydrogen-bond acceptors and its angle is more suitable for hydrogen bonding with Thr 347. Among the R2 groups, compounds containing hydrogen-bond acceptors such as furan groups are preferred. Overall, the R2 groups had a greater impact on the activity of compounds than R1 groups.

In addition, ADAM10 can mediate the first cleavage of Notch protein (Li et al., 2018; Li et al., 2020; Loganathan et al., 2020). There may be a compensatory effect between ADAM10 and ADAM17. In addition to ADAM17 and ADAM10, the second-step cleavage of Notch proteins by γ-secretase is notable (Sakamoto et al., 2021; Yang et al., 2021). Jia et al. systematically summarized small-molecule inhibitors that act on γ-secretase (Zhang et al., 2017). Hence, we will attempt to synthesize small-molecule inhibitors of ADAM17 and ADAM10 in the future.

Most important, the aim of this study was to directly and specifically design and obtain a small molecule inhibitor targeting ADAM17. We obtained the preferred compound: compound 2b, which inhibits the activity of ADAM17 to block the cleavage and activation of Notch proteins by targeting ADAM17. We have used molecular docking to verify the selectivity of the action of compound 2b on ADAMs: the interaction of compound 2b with ADAM17 is significantly stronger than that of the other ADAMs. Although the crystal structures of only four ADAMs (ADAM33, ADAM22, ADAM10, or ADAMTS-4) in addition to ADAM17 are currently crystal structures have been resolved, the results for these five ADAMs are also illustrative. Moreover, there are three existing small molecule inhibitors of ADAM17: pratastat (Bergmeier et al., 2004; Zhang et al., 2004), terfenadine (An et al., 2017; Li et al., 2019) and ZLDI-8 (Li et al., 2018; Lu et al., 2019; Lu et al., 2020; Zhang et al., 2023). Overall, the activity of compound 2b is superior to these three existing compounds. This is reflected by three results: (1) in the fact that the binding energy of compound 2b to ADAM17 is lower than that of pratastat, terfenadine and ZLDI-8; (2) and the *IC*_*50*_ values of compound 2b for inhibition of ADAM17 activity (inhibition of Notch downstream resistance factor Survivin) are lower than those of pratastat, terfenadine and ZLDI-8; (3) and the effect of compound 2b to enhance the sensitivity of NSCLC cells to Gefitinib is better compared with the pratastat, terfenadine and ZLDI-8. These results all point to the advantages of compound 2b over existing drugs.

## Data Availability

The original contributions presented in the study are included in the article/Supplementary Material, further inquiries can be directed to the corresponding author.
