# Supplementary figures and images for: Novel structured ADAM17 small-molecule inhibitor represses ADAM17/Notch pathway activation and the NSCLC cells’ resistance to anti-tumour drugs
Source: Front Pharmacol. 2023 Jun 29;14:1189245. doi: 10.3389/fphar.2023.1189245 (PMC10338884; doi:10.3389/fphar.2023.1189245)

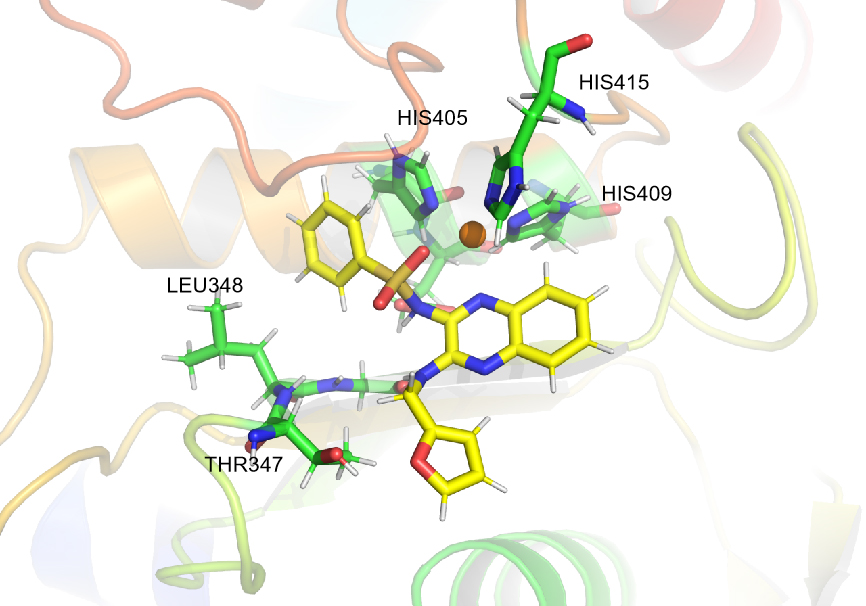

Supplement: Supplementary file 1 [file Image1.jpg]
